# Estimating prevalence of subjective cognitive decline in and across international cohort studies of aging: a COSMIC study

**DOI:** 10.1186/s13195-020-00734-y

**Published:** 2020-12-18

**Authors:** Susanne Röhr, Alexander Pabst, Steffi G. Riedel-Heller, Frank Jessen, Yuda Turana, Yvonne S. Handajani, Carol Brayne, Fiona E. Matthews, Blossom C. M. Stephan, Richard B. Lipton, Mindy J. Katz, Cuiling Wang, Maëlenn Guerchet, Pierre-Marie Preux, Pascal Mbelesso, Karen Ritchie, Marie-Laure Ancelin, Isabelle Carrière, Antonio Guaita, Annalisa Davin, Roberta Vaccaro, Ki Woong Kim, Ji Won Han, Seung Wan Suh, Suzana Shahar, Normah C. Din, Divya Vanoh, Martin van Boxtel, Sebastian Köhler, Mary Ganguli, Erin P. Jacobsen, Beth E. Snitz, Kaarin J. Anstey, Nicolas Cherbuin, Shuzo Kumagai, Sanmei Chen, Kenji Narazaki, Tze Pin Ng, Qi Gao, Xinyi Gwee, Henry Brodaty, Nicole A. Kochan, Julian Trollor, Antonio Lobo, Raúl López-Antón, Javier Santabárbara, John D. Crawford, Darren M. Lipnicki, Perminder S. Sachdev, for Cohort Studies of Memory in an International Consortium (COSMIC)

**Affiliations:** 1 Institute of Social Medicine, Occupational Health and Public Health (ISAP), Medical Faculty, University of Leipzig, Philipp-Rosenthal-Straße 55, 04103 Leipzig, Germany; 2 Global Brain Health Institute (GBHI), Trinity College Dublin, Dublin, Ireland; 3 Department of Psychiatry, University of Cologne, Medical Faculty, Cologne, Germany; 4 German Center for Neurodegenerative Diseases (DZNE), Bonn, Germany; 5 Department of Neurology, School of Medicine, Atma Jaya Catholic University of Indonesia, Jakarta, Indonesia; 6 Center for Health Research, School of Medicine, Atma Jaya Catholic University of Indonesia, Jakarta, Indonesia; 7 Cambridge Public Health, Cambridge University, Cambridge, UK; 8 MRC Biostatistics Unit, Institute of Public Health, Cambridge University, Cambridge, UK; 9 Institute of Health and Society, Newcastle University, Newcastle upon Tyne, UK; 10 Saul R. Korey Department of Neurology, Albert Einstein College of Medicine, Yeshiva University, New York, NY USA; 11 Department of Epidemiology and Population Health, Albert Einstein College of Medicine, Yeshiva University, New York, NY USA; 12 Department of Psychiatry and Behavioral Medicine, Albert Einstein College of Medicine, Yeshiva University, New York, NY USA; 13 INSERM, U1094, Tropical Neuroepidemiology, Limoges, France; 14 Univ. Limoges, U1094, Tropical Neuroepidemiology, Institute of Epidemiology and Tropical Neurology, GEIST, Limoges, France; 15 IRD, Associated Unit, Tropical Neuroepidemiology, Limoges, France; 16 CHU, Department of Medical Information & Evaluation, Clinical Research and Biostatistic Unit, Limoges, France; 17 Department of Neurology, Amitié Hospital, Bangui, Central African Republic; 18 INSERM U1061 Neuropsychiatry: Epidemiological and Clinical Research, La Colombière Hospital, Montpellier Cedex 5, Université de Montpellier, Montpellier, France; 19 Centre for Clinical Brain Sciences, University of Edinburgh, Edinburgh, UK; 20 Golgi Cenci Foundation, Corso San Martino 10, 20081 Abbiategrasso, Italy; 21 Department of Neuropsychiatry, Seoul National University Bundang Hospital, Seongnam, Republic of Korea; 22 Department of Psychiatry, Seoul National University College of Medicine, Seoul, Republic of Korea; 23 Department of Brain and Cognitive Science, Seoul National University College of Natural Sciences, Seoul, Republic of Korea; 24 Centre of Healthy Aging and Wellness, Faculty of Health Sciences, Universiti Kebangsaan Malaysia, Kuala Lumpur, Malaysia; 25 Centre for Rehabilitation Science and Special Needs, Faculty of Health Science, Universiti Kebangsaan Malaysia, Kuala Lumpur, Malaysia; 26 School of Health Science, Universiti Sains Malaysia, Kota Bharu, Kelantan Malaysia; 27 Department of Psychiatry and Neuropsychology, School for Mental Health and Neuroscience (MHeNs), Maastricht University Medical Center (MUMC), Maastricht, The Netherlands; 28 Department of Psychiatry, University of Pittsburgh School of Medicine, Pittsburgh, PA USA; 29 Department of Neurology, University of Pittsburgh School of Medicine, Pittsburgh, PA USA; 30 Department of Epidemiology, University of Pittsburgh Graduate School of Public Health, Pittsburgh, PA USA; 31 Ageing Futures Institute, University of New South Wales, Sydney, NSW Australia; 32 Neuroscience Research Australia, Sydney, NSW Australia; 33 Centre for Research on Ageing, Health and Wellbeing, College of Health and Medicine, Australian National University, Canberra, ACT Australia; 34 Center for Health Science and Counseling, Kyushu University, 744 Motooka, Nishi-ku, Fukuoka, 819-0395 Japan; 35 Department of Epidemiology and Public Health, Graduate School of Medical Sciences, Kyushu University, 3-1-1 Maidashi, Higashi-ku, Fukuoka, 812-8582 Japan; 36 Faculty of Socio-Environmental Studies, Department of Socio-Environmental Studies, Fukuoka Institute of Technology, 3-30-1 Wajiro-higashi, Higashi-ku, Fukuoka, 811-0295 Japan; 37 Department of Psychological Medicine, Yong Loo Lin School of Medicine, National University of Singapore, Singapore, Singapore; 38 Centre for Healthy Brain Ageing, School of Psychiatry, University of New South Wales Sydney, Sydney, Australia; 39 Dementia Collaborative Research Centre, School of Psychiatry, University of New South Wales Sydney, Sydney, Australia; 40 Department of Developmental Disability Neuropsychiatry, University of New South Wales Sydney, Sydney, Australia; 41 Centro de Investigación Biomédica en Red de Salud Mental (CIBERSAM), Ministry of Science and Innovation, Madrid, Spain; 42 Department of Medicine and Psychiatry, Universidad de Zaragoza and Instituto de Investigación Sanitaria Aragón, Zaragoza, Spain; 43 Department of Psychology and Sociology, Universidad de Zaragoza, Zaragoza, Spain; 44 Department of Microbiology, Preventive Medicine and Public Health, University of Zaragoza, Zaragoza, Spain

**Keywords:** Subjective cognitive decline, Prevalence, Epidemiology, Individual participant data, Data harmonization, Cohort study

## Abstract

**Background:**

Subjective cognitive decline (SCD) is recognized as a risk stage for Alzheimer’s disease (AD) and other dementias, but its prevalence is not well known. We aimed to use uniform criteria to better estimate SCD prevalence across international cohorts.

**Methods:**

We combined individual participant data for 16 cohorts from 15 countries (members of the COSMIC consortium) and used qualitative and quantitative (Item Response Theory/IRT) harmonization techniques to estimate SCD prevalence.

**Results:**

The sample comprised 39,387 cognitively unimpaired individuals above age 60. The prevalence of SCD across studies was around one quarter with both qualitative harmonization/QH (23.8%, 95%CI = 23.3–24.4%) and IRT (25.6%, 95%CI = 25.1–26.1%); however, prevalence estimates varied largely between studies (QH 6.1%, 95%CI = 5.1–7.0%, to 52.7%, 95%CI = 47.4–58.0%; IRT: 7.8%, 95%CI = 6.8–8.9%, to 52.7%, 95%CI = 47.4–58.0%). Across studies, SCD prevalence was higher in men than women, in lower levels of education, in Asian and Black African people compared to White people, in lower- and middle-income countries compared to high-income countries, and in studies conducted in later decades.

**Conclusions:**

SCD is frequent in old age. Having a quarter of older individuals with SCD warrants further investigation of its significance, as a risk stage for AD and other dementias, and of ways to help individuals with SCD who seek medical advice. Moreover, a standardized instrument to measure SCD is needed to overcome the measurement variability currently dominant in the field.

## Background

In light of the projected increase of people living with dementia all around the world, there is a strong interest in early risk stages that may allow for early intervention or the prevention of dementia [1]. Subjective cognitive decline (SCD) has recently attracted renewed attention on the assumption that it could be the first notable manifestation in the preclinical stage of Alzheimer’s disease (AD) and other dementias [2]. SCD refers to a self-experienced decline in cognitive ability in comparison with a previously normal status and without objective cognitive impairment [3]. The updated AD research framework of the National Institute on Aging and Alzheimer’s Association (NIA-AA) now recognizes SCD within the cognitively unimpaired stage on the cognitive continuum [4]. Thus, SCD is considered a risk stage for dementia. This is supported by evidence from longitudinal epidemiological data that show an increased risk for mild cognitive impairment (MCI) and dementia in individuals with SCD [2].

Importantly, the subjective perception of declining cognitive capacity can also emerge due to conditions other than AD, for example as part of normal aging, MCI, and in association with depression and anxiety [3]. Primarily, SCD is considered a symptom of preclinical AD only in association with AD biomarkers; however, another view is to consider SCD as a broader behavioral phenotype [5] above and beyond preclinical AD that defines a group of people being concerned about their brain health. This is reflected in an increasing number of individuals who seek medical advice because of SCD [6].

Despite the growing research interest in SCD, the concept still faces methodological challenges regarding its operationalization [7]. Historically, the field has lacked a common terminology and definition since the initial description of a “forgetfulness phase” by Reisberg et al. in 1982 [8]. This resulted in the dissemination of a variety of terms, e.g., subjective memory complaints, subjective memory impairment, forgetfulness, subjective cognitive impairment, or cognitive concerns—to name a few. This hurdle has recently been cleared through the introduction of a consensus definition of SCD in preclinical AD by the working group of the Subjective Cognitive Decline Initiative (SCD-I) [3]. A standard approach to measure SCD, however, is still lacking [9]. In light of the evolution of the concept, it is unsurprising that previous findings on SCD epidemiology varied, including prevalence estimates. Early studies investigating more general memory complaints reported prevalences between 22 and 56% in community-based samples [10]. Studies estimating SCD prevalence based on the SCD-I criteria are scarce, but there are some examples: In a sample representing the Greek population aged ≥ 65 years without psychiatric conditions, 28% of the cognitively unimpaired participants reported SCD [11]. In a German sample of cognitively unimpaired individuals aged ≥ 75 years, SCD prevalence was 54% [12]. In Chinese residents aged 60 to 80 years, SCD prevalence ranged between 14 and 19% [13]. This, together with a lack of standardized SCD assessment and variations in case definitions, explains variance in reported outcomes. Notably, the occurrence of SCD has almost exclusively been studied in high-income countries (HIC), while evidence from low- and middle-income countries (LMIC) is lacking. The SCD-I emphasized the need for harmonized observational studies that can attenuate some of the limitations associated with SCD operationalization [7].

### Study aims

We aimed to estimate SCD prevalence in cognitively unimpaired older individuals by applying uniform SCD criteria to harmonized data from 16 diverse cohort studies of aging. By doing so, we aimed to minimize the influence of both study level and individual level factors, thereby enhancing the generalizability of findings [14]. Moreover, as SCD prevalence may vary across subgroups, we aimed to examine differences in SCD prevalence according to sociodemographic (age, gender, education, and ethnicity) and regional (country income) factors as well as time (decade of study baseline) across studies.

## Methods

### Contributing studies and participants

Cross-sectional population-based individual participant data (IPD) were contributed by 16 member studies of the Cohort Studies of Memory in an International Consortium (COSMIC; https://cheba.unsw.edu.au/consortia/cosmic/studies) [15] (Table 1; see Supplemental material Table e-1 for key references). COSMIC brings together international cohort studies of aging to foster cross-cohort analyses on common factors for cognitive decline and dementia. Study-based data were harmonized and pooled. Baseline data were used for all studies except for two that did not assess all variables needed for SCD classification until later waves, i.e., the Monongahela Valley Independent Elders Survey (MoVIES) provided data for wave 2 (2 years after baseline) and the Personality and Total Health Through Life Project (PATH) for wave 3 (8 years after baseline). The initial sample included individuals aged at least 60 years and without a dementia diagnosis (Table e-2). Drawing on a dementia-free sample was the default option as some studies excluded prevalent dementia cases as per their study design. For the current study, data represented 15 countries from Africa, Asia, Australia, Europe, and North America.

**Table 1:** Contributing studies (*n* = 16)

Study	Abbreviation	Country	Country income group*	Baseline (years)	Sample size
Atma Jaya Cognitive & Aging Research	ActiveAging	Indonesia	LMIC	2009	278
Cognitive Function & Ageing Studies	CFAS	UK	HIC	1989–1991	12,457
Einstein Aging Study	EAS	USA	HIC	1993	2154
Epidemiology of Dementia in Central Africa	EPIDEMCA	Republic of Congo, Central African Republic	LIC/LMIC	2011–2012	1867
Etude Santé Psychologique et Traitement	ESPRIT	France	HIC	1999–2001	2190
Invecchiamento Cerebrale in Abbiategrasso	Invece.Ab	Italy	HIC	2010	1280
Korean Longitudinal Study on Cognitive Aging and Dementia	KLOSCAD	Korea	HIC	2009–2012	6430
Leipzig Longitudinal Study of the Aged	LEILA75+	Germany	HIC	1997	1045
Long-term Research Grant Scheme - Towards Useful Aging	LRGS-TUA	Malaysia	UMIC	2012–2013	2131
Maastricht Aging Study	MAAS	Netherlands	HIC	1993	500
Monongahela Valley Independent Elders Survey	MoVIES	USA	HIC	1987–1989	1276
Personality and Total Health Through Life Project	PATH	Australia	HIC	2001	1965
Sasaguri Genkimon Study	SGS	Japan	HIC	2011	2618
Singapore Longitudinal Ageing Studies II	SLASII	Singapore	HIC	2003	2585
Sydney Memory and Ageing Study	SydneyMAS	Australia	HIC	2005–2007	1037
Zaragoza Dementia Depression Project	ZARADEMP	Spain	HIC	1994	4415

*HIC* high-income country, *LIC* low-income country, *LMIC* lower-middle income country, *UMIC* upper-middle income country

*According to World Bank classification at year/mean year of baseline assessment

### Ethics

The Human Research Ethics Committee of the University of New South Wales approved this study (Ref: HC17292). All contributing studies had previously obtained approval from their respective ethics committees, and it was standard that participants provided written informed consent.

### Demographics

Information included age, gender, education, ethnicity, country income, and decade in which a study was conducted. Education was provided as years for most studies. For four cohorts (ActiveAging, Epidemiology of dementia in Central Africa/EPIDEMCA, Etude Santé Psychologique et Traitement/ESPRIT, Maastricht Aging Study/MAAS), education data were provided as categories. For harmonization purposes, categories were assigned discrete year values based on the local education system as informed by the study leaders. A fifth cohort (MoVIES) provided category data for educational levels for all participants and values for years of education for 73.4% of all participants. These data were used to calculate a mean year value for each category that was assigned to individuals missing education year data. For subgroup analysis only, years of education were re-categorized according to the UNESCO International Standard Classification of Education (ISCED) 2011 into pre-/primary education (0 to 5 years), secondary education (6 to 9 years), upper-/post-secondary education (10 to 14 years), and tertiary education (> 14 years) [16].

Ethnicity was recorded according to self-report, i.e., in an open question format in the Cognitive Function & Ageing Studies (CFAS) and according to pre-specified categories in the Einstein Aging Study (EAS), the Long-term Research Grant Scheme - Towards Useful Aging (LRGS-TUA), MoVIES, and the Sydney Memory and Ageing Study (MAS). All other studies did not assess ethnicity. Therefore, participants’ ethnicity was assigned as the majority ethnicity of the study sample as informed by the study leaders. Due to the lack of data granularity with regard to ethnic groups, analysis was based on three major ethnic groups: Asian people, Black African people, and White people.

Country income was categorized according to the World Bank classification corresponding to the year or mean year in which baseline assessments took place. It is based on the gross national income per capita, i.e., low-, lower middle-, upper middle-, and high-income country [17].

To account for a potential impact of time trends regarding dementia awareness, we furthermore considered the decade, in which studies were initiated (< 1999, 2000–2009, > 2009).

### Assessment of self-experienced decline in cognitive capacity

Assessment of a self-experienced decline in cognitive capacity varied across studies, though answers to all questions were self-reported during face-to-face interviews, using paper and pencil questionnaires (see Table e-3 for an overview of instruments). Six studies used a single self-developed question, seven studies used a battery of self-composed questions, and one study used the Subjective Memory Complaints Questionnaire (SMCQ) [18]. For two studies (ActiveAging and Long-term Research Grant Scheme - Towards Useful Aging/LRGS-TUA), the item “Do you feel you have more problems with memory than most?” from the Geriatric Depression Scale (GDS-15) was used [19]. In PATH, the Informant Questionnaire on Cognitive Decline in the Elderly (IQCODE) was administered as self-report, including seven items that addressed whether different facets of memory and recall had changed over time [20]. Data on all identified items were then synthesized and harmonized.

One fundamental challenge of integrative data analysis is the evaluation and statistical consideration of between-sample heterogeneity. Since the majority of contributing studies used multiple items to assess a self-experienced decline in cognitive capacity, we followed two complementary strategies to prepare data.

### Qualitative harmonization of items for a self-experienced decline in cognitive capacity

The first harmonization strategy followed a qualitative approach [21]. Authors SR and AP independently compared all items assessing self-experienced decline in cognitive capacity across studies and then selected and harmonized them by matching semantically similar items, i.e., if more than one item was available. Inter-rater agreement was high (*K* = 0.97). The original scales for all items were transformed, if necessary, to provide binary responses of presence or absence of a self-experienced decline in cognitive capacity. As a result, we identified and harmonized one common item that bridges the measurement of a self-experienced decline in cognitive capacity across studies (we refer to this item as “item 1”). Table e-3 shows the assessment of the selected items underlying the harmonization of item 1 across studies. Commonly, these items broadly addressed whether the study participant had problems or difficulties with memory; MAAS provided a single item assessing cognitive failures. For six studies, only data on this bridging item 1 was available. For the remaining ten studies, information on additional items was available and considered for harmonization. Overall, we were able to identify and harmonize a total of 32 different items for IRT analysis (Table e-4).

### Quantitative harmonization of items for a self-experienced decline in cognitive capacity

The second harmonization strategy followed a quantitative approach in order to develop a measurement model for both common and study-unique items. The goal was to generate scale scores for a generic construct of self-experienced cognitive decline, which is commensurate in meaning and metric across studies and study subpopulations [22]. In particular, we used the 2-Parameter Logistic (2-PL) Item Response Theory (IRT) model as a psychometric approach to evaluate measurement equivalence of items across the 16 studies. IRT allows the localization of both item difficulty and person characteristics on a common latent “trait” that represents self-experienced decline in cognitive capacity, while controlling for between-study heterogeneity of measurement. Higher difficulty values indicate a higher likelihood of a positive response to a certain item [22]. First, we applied an automated bottom-up stepwise item selection procedure to identify a core set of items that builds the basis for IRT analysis [23]. The procedure revealed that 18 of the 32 items (see Table e-4) form a unidimensional factor with acceptable scalability (Loevinger’s *H*_*ij*_ = 0.43) and good reliability (Cronbach’s Alpha = 0.81). Inspection of parameters of the fitted 2-PL IRT model (i.e., difficulty, discrimination), as well as the evaluation of item characteristics curves, led to the exclusion of two additional items with insufficient psychometric properties (item 2, item 5), resulting in a final IRT model with 16 items with partial factorial invariance and the following fit indices: root mean square error of approximation = .02; comparative fit index = .87; Tucker Lewis Index = .85; standardized root mean square residuals = .09, indicating an only approximately acceptable model fit. However, inspection of alternative models did not reveal a better fitting model across the 16 contributing studies, i.e. the presented IRT model represented the best fit. Finally, we dichotomized the predicted latent score obtained from the final IRT model at *theta* = 0 to differentiate individuals with a higher likelihood of present symptoms from those with lower estimated likelihood [24]. The cut of *theta* = 0 was pre-specified prior to the analysis as there is no gold standard to draw on and it may best separate cases from non-cases as it represents the mean of the calibration sample for the IRT model.

### Cognitive impairment

The Mini-Mental State Examination (MMSE) was used to assess cognitive impairment [25]. This was the default option as the MMSE was assessed in all but two studies, for which MMSE scores could be derived from similar tests. For the Einstein Aging Study (EAS), Blessed Information Memory Concentration scores were converted to MMSE scores using a validated formula [26, 27]. The formula was based on correlational analysis of the scores of the two tests in individuals with AD and both tests demonstrated high test-retest reliability of *r* = .75 and above, thus showing high consistency [27]. For the EPIDEMCA cohort, Community Screening Interview for Dementia (CSI ‘D’) scores were converted to MMSE scores using a co-calibration table [28, 29]. The co-calibration table was developed for four commonly used tests of global cognitive functioning, including the CSI ‘D’ and the MMSE, using IRT based on cross-sectional data from three large (*n* > 1000) community-based studies of cognitive functioning in old age [29]. Therefore, scores from one test can be directly compared to scores on the other test.

### Functional ability

Functional ability was based on the assessment of instrumental activities of daily living (IADL). Nine studies used the Lawton and Brody IADL Scale [30]. Seven studies each used a different scale, though all had large overlap in key activities (e.g., food preparation, shopping). All instruments are listed in Table e-5. For all studies, higher scores indicated higher functionality (after reverse scoring for EPIDEMCA and SydneyMAS).

### Depressive and anxiety symptomatology

All studies contributed data for depression and 12 studies for anxiety (anxiety data were not collected in ActiveAging, Leipzig Longitudinal Study of the Aged/LEILA75+, LRGS-TUA, and MoVIES). Data were harmonized as per previous COSMIC reports, with all available information considered [31, 32]. Depression was indicated by any of scale scores meeting cut-off, expert diagnosis, self-report, treatment, or use of antidepressant medication (Table e-6). Similarly, anxiety disorders were indicated by any of scale scores meeting cut-off, self-report, or the use of anxiolytics (Table e-7).

### Operationalization of SCD cases

SCD cases were uniformly defined and operationalized according to current criteria for SCD and followed recommendations on the implementation of SCD in research, i.e., endorsement of a self-experienced decline in cognitive capacity in the absence of objective cognitive impairment (criterion 1), unimpaired functional ability (criterion 2), exclusion of major depression (criterion 3), and exclusion of anxiety disorder (criterion 4) [2, 7].

Endorsement of a self-experienced decline in cognitive capacity and the presence or absence of major depression or anxiety disorder were determined as above.

Unimpaired cognitive functioning was operationalized as a score higher than 1.5 SDs below the mean of the study-based age-, gender-, and education-adjusted MMSE score in order to differentiate individuals with and without (mild) cognitive impairment. Likewise, unimpaired functional ability was operationalized as a score higher than 1.5 SDs below the mean of the study-based age-, gender-, and education-adjusted IADL scores.

### Data analysis

Prevalence in our study was defined as the number of SCD cases divided by the total number of cognitively unimpaired individuals, and estimates for both the qualitative harmonization (QH) and quantitative IRT harmonization approach are presented as percentages with 95% confidence intervals (95%CI). All estimates were stratified by study and subsequently by demographic subgroups (age, gender, education, ethnicity, country income, decade). Study-specific prevalence estimates as well as subgroup-specific prevalence estimates and corresponding tests regarding differences in education, ethnicity, country income, and decade were adjusted for age and gender, using the total sample of all 16 studies included in the analysis as the standard population. Gender differences were adjusted for age and vice versa. This allowed for the direct comparison of prevalence estimates across studies and subgroups, including different distributions of core sociodemographic variables [33]. Subgroup comparisons of proportions were tested using Pearson’s chi-square test with Rao/Scott correction. Overall prevalence estimates across studies are reported as unstandardized, since data on standard populations stratified by age and gender were not available.

To illustrate the impact of the individual criteria to quantify SCD cases, we show frequency rates criteria-wise, i.e., cumulatively applying the four criteria for SCD.

A range of sensitivity analyses were performed. First, an additional analysis was conducted with regard to IRT results by excluding studies with only the bridging item (“item 1”) available. Second, we inspected the contingency of the results of the two harmonization strategies (QH and IRT). Lastly, as the MMSE scores may have limited sensitivity and specificity to differentiate unimpaired cognitive performance from MCI [7], we conducted a sensitivity analysis with a subset of studies (*n* = 10) that were able to provide neuropsychological test scores on at least three out of four cognitive domains (memory, language, processing speed and executive function; see Table e-9 for an overview of contributing studies and neuropsychological tests; harmonization procedure as in previous COSMIC studies [31, 32]). To establish unimpaired cognitive performance based on the cognitive domains scores, the first step was to adjust test scores for age, sex, and education, and for all interactions between these variables using regression analyses within each study. The adjusted test scores were then transformed to *z*-scores using the mean and SD of the study sample as normative values. Cognitive impairment for each cognitive score was defined as performance of more than 1.5 SDs below the mean within the relevant study’s sample. To be classified cognitively impaired, this had to be the case in at least one of the considered domains. Otherwise, operationalization of SCD and statistical analysis was identical to the MMSE-based procedure.

Analyses were performed in Stata 15 MP (Stata Corp, College Station, TX) and R version 3.5.0 [34].

## Results

### Sample

The initial sample comprised 44,228 dementia-free individuals aged at least 60 years. Inspection of the initial sample led to the exclusion of 1037 (2.3%) individuals due to study-based missing information on any of the 32 SCD items and 1049 (2.4%) individuals due to missing information on demographics and/or MMSE. In addition, 2755 (6.2%) individuals classified as cognitively impaired were excluded, resulting in an analytical sample of 39,387 individuals (Table 2). The mean age of participants in the analytical sample was 73.1 years (*SD* = 7.1; range = 60–105 years). The proportion of women was 57.7%. Education was 9.1 years (*SD* = 4.4) on average.

**Table 2:** Baseline characteristics of the analytical sample (*n* = 39,387)

Study	Country	Sample size	Age ***M*** (***SD***; ***R***)	Females (***%***)	Education in years ***M*** (***SD***)	Main ethnicity
ActiveAging	Indonesia	260	68.60 (7.66; 60–97)	64.2	6.21 (4.73)	Asian
CFAS	UK	11,363	74.82 (6.64; 64–105)	59.2	10.03 (2.28)	White
EAS	USA	1950	78.53 (5.34; 64–100)	61.3	13.26 (3.59)	White (66.6%), African American/Black (27.0%)
EPIDEMCA	Republic of Congo, Central African Republic	1736	72.74 (6.44; 65–99)	60.4	2.06 (3.86)	Black African
ESPRIT	France	2022	73.09 (5.50; 65–96)	58.0	12.34 (2.26)	White
Invece.Ab	Italy	1142	72.15 (1.28; 70–75)	53.9	7.00 (3.27)	White
KLOSCAD	Korea	5784	69.78 (6.48; 60–96)	55.7	8.37 (5.29)	Asian
LEILA75+	Germany	875	81.51 (4.87; 75–99)	73.6	11.95 (1.81)	White
LRGS-TUA	Malaysia	1964	68.60 (5.94; 60–92)	50.2	5.47 (3.97)	Asian
MAAS	Netherlands	457	68.94 (6.02; 60–83)	49.5	9.47 (2.82)	White
MoVIES	USA	1188	74.06 (5.35; 66–97)	60.4	11.30 (2.48)	White
PATH	Australia	1469	70.60 (1.49; 68–74)	49.7	14.08 (2.67)	White
SGS	Japan	1948	73.44 (6.05; 65–93)	58.0	11.10 (2.50)	Asian
SLASII	Singapore	2364	69.02 (6.71; 60–94)	60.7	5.41 (4.17)	Asian
SydneyMAS	Australia	966	78.85 (4.82; 70–91)	55.2	11.63 (3.49)	White
ZARADEMP	Spain	3899	73.15 (9.20; 60–102)	56.5	7.18 (3.83)	White
**Total**	**N/A**	**39,387**	**73.07 (7.08; 60–105)**	**57.7**	**9.13 (4.43)**	**N/A**

*M* mean, *N/A* not applicable, *R* range, *SD* standard deviation

### Prevalence of SCD in and across studies

Overall, both the QH and IRT approach robustly suggested a SCD prevalence of roughly one in four in the older population without cognitive impairment across studies (QH 23.8% [95%CI = 23.3–24.4%]; IRT 25.6% [95%CI = 25.1–26.1%]). Study-based age- and gender-standardized SCD prevalence estimates varied largely, ranging from 6.1% (95%CI = 5.1–7.0%) to 52.7% (95%CI = 47.4–58.0%) for QH and 7.8% (95%CI = 6.8–8.9%) to 52.7% (95%CI = 47.4–58.0%) for IRT. All study-based SCD prevalence estimates according to QH and IRT are shown in the last column in Tables 3 and 4, respectively.

**Table 3:** Study-based age- and gender-standardized prevalence estimates for subjective cognitive decline (SCD; last column) and cumulative frequency estimates for the stepwise application of SCD operationalization criteria according to qualitative harmonization

Study	SCD operationalization criteria			
	Criterion 1 Endorsement of a self-experienced decline in cognitive capacity without objective cognitive impairment	+ Criterion 2 Intact functional ability	+ Criterion 3 No major depression	+ Criterion 4 No anxiety disorder
	% (95%CI)			
ActiveAging	27.98 (21.44–34.52)	25.81 (19.41–32.20)	25.73 (19.36–32.10)	N/A
CFAS	17.04 (15.45–18.64)	15.59 (14.00–17.18)	12.73 (11.41–14.05)	6.34 (5.57–7.20)
EAS	15.84 (12.57–19.10)	13.82 (10.90–16.73)	15.23 (10.95–19.51)	12.05 (5.80–18.30)
EPIDEMCA	50.52 (48.36–52.69)	47.74 (45.51–49.98)	25.83 (23.80–27.86)	23.86 (21.93–25.79)
ESPRIT	18.17 (16.52–19.82)	16.60 (15.02–18.18)	13.36 (11.92–14.80)	10.35 (9.11–11.60)
Invece.Ab	14.60 (11.63–17.56)	14.02 (10.89–17.16)	12.31 (9.43–15.18)	10.76 (8.28–13.23)
KLOSCAD	63.65 (62.28–65.02)	58.02 (57.88–59.46)	42.25 (40.82–43.68)	42.27 (40.90–43.66)
LEILA75+	12.88 (11.65–14.12)	11.70 (10.48–12.92)	9.80 (8.64–10.96)	N/A
LRGS-TUA	51.23 (48.15–54.31)	47.39 (44.32–50.47)	46.84 (43.80–49.87)	N/A
MAAS	65.26 (60.33–70.20)	60.52 (55.35–65.69)	54.97 (49.43–60.50)	52.70 (47.43–57.96)
MoVIES	32.82 (30.35–35.32)	30.45 (28.01–32.90)	27.27 (24.89–29.65)	N/A
PATH	8.95 (7.89–10.01)	8.23 (7.21–9.26)	7.34 (6.36–8.33)	6.06 (5.13–6.99)
SGS	18.47 (16.82–20.13)	16.52 (14.93–18.10)	10.24 (8.94–11.55)	9.91 (8.64 (11.18)
SLASII	12.16 (10.53–13.79)	10.82 (9.27–12.36)	10.28 (8.78–11.78)	9.89 (8.50–11.28)
SydneyMAS	42.77 (40.49–45.04)	39.10 (36.70–41.51)	32.58 (30.14–35.01)	26.98 (24.55–29.40)
ZARADEMP	44.68 (42.98–46.38)	42.04 (40.31–43.77)	29.42 (27.77–31.06)	28.12 (26.50–29.74)
Total*	**33.03 (32.56–33.49)**	**30.46 (30.01–30.92)**	**24.08 (23.65–24.51)**	**23.83 (23.29–24.36)**

*95%CI* 95% confidence interval, *N/A* not available

*Unstandardized

**Table 4:** Study-based age- and gender-standardized prevalence estimates for subjective cognitive decline (SCD; last column) and cumulative frequency estimates for the stepwise application of SCD operationalization criteria according to quantitative harmonization/Item Response Theory (IRT) modeling

Study	SCD operationalization criteria			
	Criterion 1 Endorsement of a self-experienced decline in cognitive capacity without objective cognitive impairment	+ Criterion 2 Intact functional ability	+ Criterion 3 No major depression	+ Criterion 4 No anxiety disorder
	Prevalence estimates in % (95%CI)			
ActiveAging	27.97 (21.43–34.51)	25.80 (19.40–32.20)	25.73 (19.36–32.10)	N/A
CFAS	23.44 (21.77–25.12)	21.59 (19.91–23.26)	17.78 (16.36–19.20)	9.60 (8.63–10.58)
EAS	50.41 (45.20–55.62)	46.06 (41.06–51.06)	38.71 (34.07–43.34)	25.91 (19.33–32.48)
EPIDEMCA	56.28 (54.18–58.38)	53.02 (50.83–55.21)	30.64 (28.51–32.77)	28.41 (26.38–30.44)
ESPRIT	31.26 (29.34–33.18)	28.76 (26.89–30.63)	23.97 (22.20–25.74)	18.94 (17.36–20.51)
Invece.Ab	14.61 (11.64–17.58)	14.03 (10.90–17.17)	12.32 (9.44–15.20)	10.77 (8.28–13.26)
KLOSCAD	55.51 (54.09–56.92)	50.55 (49.10–52.00)	36.01 (34.62–37.40)	35.97 (34.62–37.31)
LEILA75+	12.89 (11.66–14.13)	11.71 (10.49–12.94)	9.81 (8.65–10.97)	N/A
LRGS-TUA	51.23 (48.15–54.31)	47.40 (44.32–50.47)	46.84 (43.80–49.87)	N/A
MAAS	65.26 (60.32–70.20)	60.52 (55.34–65.69)	54.97 (49.43–60.51)	52.70 (47.43–57.98)
MoVIES	29.39 (26.97–31.81)	27.20 (24.83–29.57)	23.87 (21.59–26.15)	N/A
PATH	11.68 (10.50–12.86)	10.76 (9.62–11.90)	9.63 (8.54–10.73)	7.83 (6.80–8.87)
SGS	18.47 (16.81–20.12)	16.51 (14.93–18.09)	10.24 (8.93–11.54)	9.91 (8.64–11.18)
SLASII	25.48 (23.37–27.59)	22.87 (20.83–24.91)	22.17 (20.16–24.18)	21.18 (19.31–23.05)
SydneyMAS	45.81 (43.41–46.39)	42.72 (40.48–44.95)	35.87 (33.55–38.19)	29.88 (27.54–32.22)
ZARADEMP	44.69 (42.99–46.39)	42.04 (40.32–43.77)	30.64 (28.51–32.77)	28.12 (26.50–29.74)
Total*	**37.32 (36.85–37.79)**	**34.49 (34.02–34.97)**	**27.75 (27.31–28.20)**	**25.60 (25.06–26.14)**

*95%CI* 95% confidence interval, *N/A* not available

*Unstandardized

### Proportions according to cumulative criteria application

The columns of Tables 3 and 4 show the proportions of the incremental application of the four operationalization criteria for SCD. There was a particular difference in proportions between functional criteria (endorsement of a self-experienced decline in cognitive capacity without objective cognitive impairment and functional ability) and mood criteria (depressive and anxiety symptomatology). When in addition to functional criteria depressive and anxiety symptomatology were addressed, overall proportions decreased from 30.5 to 23.8% (95%CI = 30.0–30.9%; 95%CI = 23.3–24.4%, respectively) in QH and from 34.5 to 25.6% (95%CI = 34.0–35.0%; 95%CI = 25.1–26.1%, respectively) in IRT.

### Prevalence of SCD according to age group and gender across studies

**Fig. 1:**
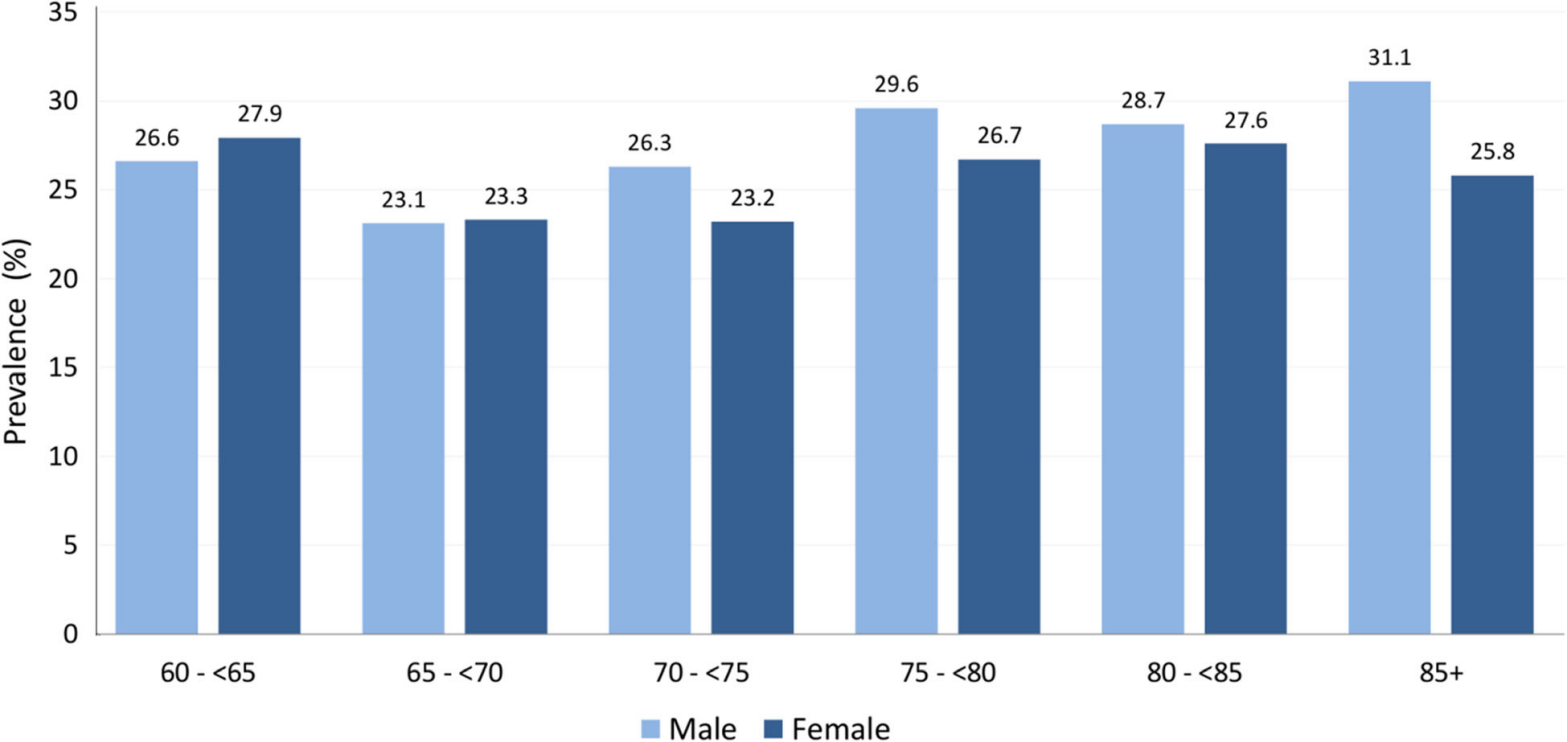
SCD prevalence according to age groups and gender in older individuals (≥ 60 years) without cognitive impairment across international cohort studies (percentages). Estimates are based on Item Response Theory (IRT) analysis

The following subgroup results are based on IRT analysis, applying all four SCD criteria. Gender differences were adjusted for age and vice versa. The subgroup results of the qualitative approach are detailed in Table e-10. Overall, SCD prevalence was somewhat higher in men compared to women (26.6% [95%CI = 25.7–27.4%] vs. 24.9% [95%CI = 24.2–25.6%]; *χ*^2^(1) = 8.54; *p* = .003). SCD prevalence significantly differed according to age group (*χ*^2^(5) = 51.31; *p* < .001), which also applied when stratified for gender (men: *χ*^2^(5) = 32.42; *p* < .001; women: *χ*^2^(5) = 29.81; *p* < .001). SCD prevalence was 27.4% (95%CI = 25.8–28.9%) in ages 60–64 years, then decreased to 23.2% (95%CI = 22.1–24.2%) in ages 65–69 years, thereafter increased to 24.5% (95%CI = 23.6–25.5%) in ages 70–74 years, 27.9 (95%CI = 26.5–29.3%) in ages 75–79 years, and 28.1% (95%CI = 26.2–30.0%) in ages 80–84 years and 28.1% (95%CI = 25.7–30.9%) in ages 85+ years. Overall, there was no clear pattern associated with age, except for those aged 65–74 years generally having a lower prevalence than all other age groups. Figure 1 additionally shows SCD prevalence according to age groups stratified by gender.

### Prevalence of SCD according to education across studies

Age- and gender-standardized SCD prevalence significantly differed according to level of education (*χ*^2^(3) = 68.37; *p* < .001). Individuals with pre-/primary education had higher a prevalence of SCD (29.0%; 95%CI = 27.9–30.2%) compared to individuals with secondary education (25.0%; 95%CI = 24.1–25.9%), secondary upper/post education (22.7%; 95%CI = 21.8–23.8%), and tertiary education (26.4%; 95%CI = 24.8–28.0%).

### Prevalence of SCD according to ethnicity across studies

Due to missing information on self-reported ethnicity, this subgroup analysis was based on a subsample of *n* = 23,641 individuals. Age- and gender-standardized SCD prevalence in White people (24.3%; 95%CI = 23.5–25.1%) was significantly lower than in Asian people (27.2%; 95%CI = 26.3–28.1%) and Black African people (28.2%; 95%CI = 26.2–30.3%; *Χ*^2^(2) = 29.39; *p* < .001). Data availability did not allow including further ethnic groups.

### Prevalence of SCD according to country income across studies

Age- and gender-standardized SCD prevalence was significantly associated with country income (*χ*^2^(2) = 9.56; *p* < .001). The lower the country income, the higher the SCD prevalence, increasing from 25.1% (95%CI = 24.5–25.6%) in HIC to 27.4% (95%CI = 24.6–30.5%) in LMIC to 29.3% (95%CI = 26.4–32.3%) in LIC.

### Prevalence of SCD according to decade of study conduction

Age- and gender-standardized SCD prevalence tended to differ by decade (*χ*^2^(2) = 47.77; *p* < .001), showing an increase over time from 22.4% (95%CI = 21.4–23.3%) before 1999, then 24.5% (95%CI = 23.1–25.9%) in the years 2000–2009 to 26.3% (95%CI = 25.9–27.4%) after 2009.

### Sensitivity analysis

Results of the IRT-based sensitivity analyses of only studies that used multiple items to assess a subjective experience of cognitive decline are shown in Table e-8, further supporting a pooled SCD prevalence of one in four (26.1%; 95%CI = 25.4–26.7%).

The contingency of the results returned by the two harmonization approaches was continuously high across prevalence estimates. Using all four operationalization criteria, SCD case classification based on “item 1” by QH and IRT corresponded in 91.4% (*Φ* = 0.77) and in studies with multiple items in 88.0% (*Φ* = 0.68) of all cases vs. non-cases.

Sensitivity analysis utilizing cognitive domain scores instead of MMSE scores to define unimpaired cognitive function was based on a subset of ten studies. Results of the qualitative approach are presented in Table e-11 and of the quantitative/IRT approach in Table e-12. Table e-13 juxtaposes SCD prevalence estimates across studies for both the MMSE-based and cognitive domain scores-based operationalization of unimpaired cognitive performance (quantitative approach: 1.48% diff; qualitative approach: 0.80% diff.)

## Discussion

We estimated the prevalence of subjective cognitive decline (SCD) by applying uniform criteria to harmonized individual participant data (IPD). Data represented 16 international population-based cohort studies from 15 countries with over 39,000 individuals at least 60 years old. Across studies, qualitative harmonization (QH) and quantitative harmonization using Item Response Theory (IRT) both robustly suggested a SCD prevalence of roughly one quarter (23.8% and 25.6%) in cognitively unimpaired older individuals. Still, prevalence estimates varied largely between studies (QH 6.1–52.7%; IRT 7.8–52.7%). However, applying uniform criteria for SCD operationalization to harmonized data helps to increase comparability of estimates across studies. This is a strength of our study as SCD assessment greatly differed between cohorts and different measurements are known to be associated with outcome variance [35]. Indeed, differences in prevalence estimates are directly associated with test accuracy, and the majority of the items the cohorts used to measure self-experienced decline in cognitive capacity were not psychometrically evaluated. Used in isolation, these potentially imperfect items may give false-positive or false-negative results, resulting in biased prevalence estimates. As the original studies contributing to this work did not previously inspect SCD prevalence in their samples, we cannot, however, determine whether the application of uniform criteria to harmonized data would have reduced SCD prevalence variation between cohorts. A previous COSMIC study on MCI prevalence using such methods suggested this is the case [36]. Additionally, IRT analysis allowed heterogeneity between studies to be addressed statistically, by selecting a set of items that form a unidimensional scale, thus giving confidence that all items measured one construct. Hence, our study suggests that the prevalence of SCD in cognitively unimpaired older individuals is banded around 25%, which may give a more accurate idea of SCD prevalence than estimates of single studies, which are potentially more heavily influenced by measurement issues.

Our results support the notion that SCD is frequent in old age. It is most likely that only a proportion of these cases reflect pathological change due to AD or other dementias. The list of non-neurodegenerative conditions that can lead to subjective decline in cognition is long, including, apart from major depression and anxiety, normal aging, various psychiatric, neurologic and medical disorders, substance abuse and medication, as well as personality and cultural factors [3]. The current SCD research definition cannot rule out all other causes, and this is likely reflected in the range of prevalence estimates we found. In this regard, it is interesting to note the difference it made when major depression and anxiety disorders were considered as exclusion criteria in addition to cognitive impairment and functional inability (which some studies have only used), reducing estimates from roughly one third to one quarter. While the exclusion of depression and anxiety disorder is accepted in the criteria, it does highlight the impact criteria can have on prevalence estimates. In addition, many of the above named conditions are associated with an increased risk for cognitive decline and dementia themselves. Vice versa, specific symptoms, especially depressive and anxiety symptoms, can also be a consequence of neuropathological change or co-occur, so that the relationship between SCD, these conditions, and neurodegeneration is more complex [3]. In this regard, it seems useful to explore SCD in conjunction with other behavioral symptoms [37]. Like SCD, problems with mood, anxiety, drive, perception, sleep, appetite, agitation, and aggression can be precursors to cognitive decline and dementia, as summarized in the construct mild behavioral impairment (MBI) [38]. Future investigations could link the two behavioral concepts rather than studying them independently.

Age- and gender-standardized SCD prevalence differed regarding sociodemographic factors. We found slightly higher SCD prevalence in men compared to women. The literature on gender differences in SCD prevalence is inconsistent, some reporting higher prevalence in women [39, 40], others in men [41], or no difference [42]. For both men and women, SCD prevalence differed by age; however, without a clear pattern. This is similar to MCI prevalence, but different to dementia prevalence that shows an increase with aging [36].

SCD prevalence was highest in individuals with less education, which is in line with a previous study [43]. Hao et al. suggested that low education may be a risk factor for SCD, being associated with a higher likelihood of progression to MCI [13]. While the prevalence decreased with increasing levels of education, there was a tendency to increase again among those with the highest education levels. In general, higher educational attainment is thought to provide resilience against neuropathology [44]. This could be expected to lead to a delay in symptom onset and therefore, potentially, a lower SCD prevalence associated with higher levels of education. However, our finding of higher SCD prevalence in those with the highest education suggests otherwise and possibly points to increased awareness of or alertness to subtle cognitive changes in this group.

Regarding ethnicity, we found higher SCD prevalence in Asian and Black African people compared to White people, which is supposedly associated with country income as ethnic group definition was very broad due to a lack of ethnic data granularity across studies. With regard to results from other studies, a US study reported similar levels of SCD for African American people and White people [45], whereas in another US study SCD was lower in Asian people compared to White people and highest in Black American people and American Indian people [43]. The results of our study, however, should be interpreted with caution as data on ethnicity were limited and may not be representative. SCD prevalence and ethnicity is otherwise a largely unexplored topic.

SCD prevalence was lower in HIC compared to LMIC. From an ecobiopsychosocial perspective, this supports the notion that environments with higher socioeconomic resources (e.g., better health care, better educational opportunities, better lifestyle infrastructure) may be beneficial for population health [46]. Indeed, dementia incidence has been observed to have slightly declined in recent decades in Western high-income countries, supporting such an assumption [47]. Opposed to that, we found a trend of increasing SCD prevalence over decades from before 1999 to after 2009. On the one hand, this could be attributed to increasing public health awareness regarding dementia, or, on the other hand, to the fact, that latter data included more studies from LMIC whereas early studies were exclusively from HIC. However, as much of the increase in numbers of people living with dementia takes place in LMIC [48], there may be indeed a trend towards increasing SCD.

Regardless of causes or consequences, SCD is a serious issue for individuals who experience it. SCD has been associated with concerns [49], lower health-related quality of life [50], increased help-seeking behavior and health care utilization [51]. Thus, SCD has a negative impact on the individual, but also on society through creating additional costs [52]. Having roughly one quarter of the cognitively unimpaired older population experiencing SCD poses the question “what to do about it?.” A systematic review and meta-analysis of interventions for SCD targeting well-being, meta-cognition, and/or objective cognitive performance reported a lack of high-quality research, but nevertheless found that psychological interventions may be beneficial for well-being and meta-cognition in SCD, though not for cognitive performance [52]. Furthermore, the same study reported a lack of evidence regarding lifestyle and pharmacological interventions. The SCD-I recently argued in support of tailored diagnostic processes that identify underlying medical conditions in individuals with SCD who present to physicians [6]. If no cause can be identified, they suggest to inform about SCD and dementia risk. From there, a watch and wait strategy could be adopted. A comprehensive approach to deal with SCD, if no treatable underlying condition can be identified, is perhaps education about modifiable health and lifestyle factors for brain health. Increasing evidence highlights that, among other factors, improved management of diabetes, hypertension, and obesity, as well as proactive lifestyle behaviors regarding physical, cognitive, and social activity, can promote brain health and may mitigate dementia risk [53].

### Limitations

We were able to uniformly operationalize SCD across studies according to current guidelines on SCD definition criteria [3, 7]; however, there may be factors not considered in these criteria that influence prevalence estimates of SCD. From this perspective, our reported SCD prevalence may be an overestimate. The MMSE has been criticized for having limited sensitivity and specificity in differentiating between unimpaired and mildly cognitively impaired performance [7]. Thus, SCD prevalence estimates based on cognitive performance derived from MMSE scores could lead to an underestimation of prevalence. Therefore, we performed a sensitivity analysis based on a subset of studies that were able to provide more comprehensive neuropsychological test scores. In comparison, SCD prevalence estimates across studies hardly differed, which strengthened the confidence in the prevalence estimates utilizing MMSE scores. However, where possible, preference should be given to sensitive and specific tests.

Regarding IRT-based estimation of prevalence, we were not able to pre-specify a cutoff other than *theta* = 0 to differentiate between SCD cases and non-cases, as a gold standard was not available. Moreover, fit indices of our IRT model indicated room for improvement, which should be considered when possibly developing a standardized measurement of SCD. Though we were able to methodologically tackle heterogeneity in SCD measurement across studies, estimates for individual studies are likely influenced by the type of questions asked. This could be one of the reasons for the large differences in prevalence across the studies, and calls for a standardized and psychometrically sound instrument for SCD. Also, many items to assess a self-experience in cognitive capacity did not cover perceptions of change over time; the majority instead asked about current problems with memory—an acknowledged limitation in SCD research. Future studies may also explore how study-based characteristics beyond age and gender contribute to SCD prevalence variation across studies. Ultimately, only standardized and valid SCD measurement will overcome these limitations, and the SCD field should focus on the development of such an instrument. Our IRT-based item analysis can provide useful information for this. For now, the comparable results from two complimentary approaches to estimate SCD prevalence across a set of diverse studies, further supported by similar results from sensitivity analyses, provide a more accurate picture of SCD occurrence.

## Conclusions

One in four cognitively unimpaired individuals above 60 years of age is estimated to experience and report SCD. However, SCD is likely to indicate a pre-stage of AD or other dementing disorders in only the minority of cases. Nevertheless, the frequent occurrence of SCD warrants further research of its significance for dementia, and, importantly, on ways to manage SCD in clinical practice. The development and application of a standardized measure to assess SCD is imperative to further our understanding of SCD.

## Supplementary information

**Additional file 1.**

## Data Availability

Data and material in relation to this study are available for researchers from the corresponding author upon reasonable request.

## Abbreviations

2-PL: 2 Parameter Logistic Model
95%CI: 95% confidence interval
AD: Alzheimer’s disease
COSMIC: Cohort Studies of Memory in an International Consortium
EAS: Einstein Aging Study
EPIDEMCA: Epidemiology of Dementia in Central Africa ESPRIT Etude Santé Psychologique et Traitement
GDS: Geriatric Depression Scale
HIC: High-income country
IADL: Instrumental activities of daily functioning
ISCED: International Standard Classification of Education
IPD: Individual participant data
IQCODE: Informant Questionnaire on Cognitive Decline in the Elderly
IRT: Item Response Theory
LEILA75+: Leipzig Longitudinal Study of the Aged
LMIC: Low- and middle-income countries
LRGS-TUA: Long-term Research Grant Scheme - Towards Useful Aging
MAAS: Maastricht Aging Study
MBI: Mild Behavioral Impairment
MCI: Mild Cognitive Impairment
MMSE: Mini-Mental State Examination
MoVIES: Monongahela Valley Independent Elders Survey
NIA-AA: National Institute on Aging and Alzheimer’s Association
PATH: Personality and Total Health Through Life Project
QH: Qualitative Harmonization
SCD: Subjective Cognitive Decline
SCD-I: Subjective Cognitive Decline-Initiative
SD: Standard Deviation
SMCQ: Subjective Memory Complaints-Questionnaire
SydneyMAS: Sydney Memory and Ageing Study
UNESCO: United Nations Educational, Scientific and Cultural Organization

## References

[1] Alzheimer’s Disease International: World Alzheimer Report 2016 - Improving healthcare for people living with dementia: Coverage, quality and costs now and in the future. https://www.alzint.org/u/WorldAlzheimerReport2016.pdf. Accessed 5 Nov 2020.

[2] Slot RER, Sikkes SAM, Berkhof J (2019). Subjective cognitive decline and rates of incident Alzheimer’s disease and non-Alzheimer’s disease dementia. Alzheimers Dement.

[3] Jessen F, Amariglio RE, van Boxtel M (2014). A conceptual framework for research on subjective cognitive decline in preclinical Alzheimer’s disease. Alzheimers Dement.

[4] Jack CR, Bennett DA, Blennow K (2018). NIA-AA Research Framework: toward a biological definition of Alzheimer’s disease. Alzheimers Dement.

[5] Rabin LA, Smart CM, Amariglio RE (2017). Subjective cognitive decline in preclinical Alzheimer’s disease. Annu Rev Clin Psychol.

[6] Jessen F, Amariglio RE, Buckley RF (2020). The characterisation of subjective cognitive decline. Lancet Neurol.

[7] Molinuevo JL, Rabin LA, Amariglio R (2017). Implementation of subjective cognitive decline criteria in research studies. Alzheimers Dement.

[8] Reisberg B, Ferris SH, de Leon MJ, Crook T (1982). The Global Deterioration Scale for assessment of primary degenerative dementia. Am J Psychiatry.

[9] Rabin LA, Smart CM, Crane PK (2015). Subjective cognitive decline in older adults: an overview of self-report measures used across 19 international research studies. J Alzheimers Dis.

[10] Jonker C, Geerlings MI, Schmand B (2000). Are memory complaints predictive for dementia? A review of clinical and population-based studies. Int J Geriatr Psychiatry..

[11] Vlachos GS, Cosentino S, Kosmidis MH (2019). Prevalence and determinants of subjective cognitive decline in a representative Greek elderly population. Int J Geriatr Psychiatry..

[12] Roehr S, Riedel-Heller SG, Kaduszkiewicz H (2019). Is function in instrumental activities of daily living a useful feature in predicting Alzheimer’s disease dementia in subjective cognitive decline?. Int J Geriatr Psychiatry..

[13] Hao L, Wang X, Zhang L (2017). Prevalence, risk factors, and complaints screening tool exploration of subjective cognitive decline in a large cohort of the Chinese population. J Alzheimers Dis.

[14] Thompson A (2009). Thinking big: large-scale collaborative research in observational epidemiology. Eur J Epidemiol.

[15] Sachdev PS, Lipnicki DM, Kochan NA, et al. COSMIC (Cohort Studies of Memory in an International Consortium): an international consortium to identify risk and protective factors and biomarkers of cognitive ageing and dementia in diverse ethnic and sociocultural groups. 2013;13:165–BMC Neurol. 10.1186/1471-2377-13-165 PMC3827845 24195705

[16] Schneider SL. The International Standard Classification of Education 2011. In: Class and Stratification Analysis. p. 365–79.

[17] Fantom N (2016). Serajuddin U.

[18] Youn JC, Kim KW, Lee DY (2009). Development of the subjective memory complaints questionnaire. Dement Geriatr Cogn Disord.

[19] Sheikh JI, Yesavage JA (1986). Geriatric Depression Scale (GDS): recent evidence and development of a shorter version. Clin Gerontol.

[20] Jorm AF, Jacomb PA (1989). The Informant Questionnaire on Cognitive Decline in the Elderly (ICODE): socio-demographic correlates, reliability, validity and some norms. Psychol Med.

[21] Gatz M, Reynolds CA, Finkel D, Hahn CJ, Zhou Y, Zavala C (2015). Data harmonization in aging research: not so fast. Exp Aging Res.

[22] Bauer DJ, Hussong AM (2009). Psychometric approaches for developing commensurate measures across independent studies: traditional and new models. Psychol Methods.

[23] Straat JH, van der Ark LA, Sijtsma K (2013). Comparing optimization algorithms for item selection in Mokken scale analysis. J Classif.

[24] Raykov T, Marcoulides GA (2018). A course in item response theory and modeling with Stata.

[25] Folstein MF, Folstein SE, McHugh PR (1975). “Mini-mental state”. A practical method for grading the cognitive state of patients for the clinician. J Psychiatr Res.

[26] Blessed G, Tomlinson BE, Roth M (1968). The association between quantitative measures of dementia and of senile change in the cerebral gray matter of elderly subjects. Br J Psychiatry.

[27] Thal LJ, Grundman M, Golden R (1986). Alzheimer’s disease: a correlational analysis of the Blessed Information-Memory-Concentration Test and the Mini-Mental State Exam. Neurology..

[28] Hall KS, Gao S, Emsley CL, Ogunniyi AO, Morgan O, Hendrie HC (2000). Community screening interview for dementia (CSI ‘D’); performance in five disparate study sites. Int J Geriatr Psychiatry.

[29] Crane PK, Narasimhalu K, Gibbons LE (2008). Item response theory facilitated cocalibrating cognitive tests and reduced bias in estimated rates of decline. J Clin Epidemiol.

[30] Lawton MP, Brody EM (1969). Assessment of older people: self-maintaining and instrumental activities of daily Living1. The Gerontologist.

[31] Lipnicki DM, Crawford JD, Dutta R (2017). Age-related cognitive decline and associations with sex, education and apolipoprotein E genotype across ethnocultural groups and geographic regions: a collaborative cohort study. PLoS Med.

[32] Lipnicki DM, Makkar SR, Crawford JD (2019). Determinants of cognitive performance and decline in 20 diverse ethno-regional groups: a COSMIC collaboration cohort study. PLoS Med.

[33] van Belle G, Fisher L. Biostatistics: a methodology for the health sciences. 2nd ed. Hoboken: Wiley; 2004. Wiley series in probability and statistics.

[34] Team RC (2007). R: a language and environment for statistical computing.

[35] Grabe M. Measurement uncertainties in science and technology. Berlin, Heidelberg: Springer-Verlag Berlin Heidelberg, 2005.

[36] Sachdev PS, Lipnicki DM, Kochan NA (2015). The prevalence of mild cognitive impairment in diverse geographical and Ethnocultural regions: the COSMIC collaboration. PLoS One.

[37] Donovan NJ, Amariglio RE, Zoller AS (2014). Subjective cognitive concerns and neuropsychiatric predictors of progression to the early clinical stages of Alzheimer disease. Am J Geriatr Psychiatry.

[38] Ismail Z, Smith EE, Geda Y, Sultzer D, Brodaty H, Smith G (2016). Neuropsychiatric symptoms as early manifestations of emergent dementia: provisional diagnostic criteria for mild behavioral impairment. Alzheimers Dement.

[39] Kim J-M, Stewart R, Shin I-S, Choi S-K, Yoon J-S (2003). Subjective memory impairment, cognitive function and depression—a community study in older Koreans. Dement Geriatr Cogn Disord.

[40] Müller-Gerards D, Weimar C, Abramowski J (2019). Subjective cognitive decline, APOE ε4, and incident mild cognitive impairment in men and women. Alzheimers Dement: DADM.

[41] Wang L, van Belle G, Crane PK (2004). Subjective memory deterioration and future dementia in people aged 65 and older. J Am Geriatr Soc.

[42] Buckley R, Saling MM, Ames D (2013). Factors affecting subjective memory complaints in the AIBL aging study: biomarkers, memory, affect, and age. Int Psychogeriatr.

[43] Taylor CA, Bouldin ED, McGuire LC (2018). Subjective cognitive decline among adults aged ≥45 years - United States, 2015-2016. MMWR Morb Mortal Wkly Rep.

[44] Stern Y, Arenaza-Urquijo EM, Bartrés-Faz D, et al. Whitepaper: defining and investigating cognitive reserve, brain reserve, and brain maintenance. Alzheimers Dement. 2018. 10.1016/j.jalz.2018.07.219 [Epub ahead of print]. 10.1016/j.jalz.2018.07.219 PMC6417987 30222945

[45] Jackson JD, Rentz DM, Aghjayan SL (2017). Subjective cognitive concerns are associated with objective memory performance in Caucasian but not African-American persons. Age Ageing.

[46] Braveman P, Gottlieb L (2014). The social determinants of health: it’s time to consider the causes of the causes. Public Health Rep.

[47] Roehr S, Pabst A, Luck T, Riedel-Heller SG (2018). Is dementia incidence declining in high-income countries? A systematic review and meta-analysis. Clin Epidemiol.

[48] Prince M, Wimo AGM, Ali GC, Wu YT, Prina M (2015). World Alzheimer Report 2015: the global impact of dementia: an analysis of prevalence, incidence, cost and trends.

[49] Mendonca MD, Alves L, Bugalho P (2016). From subjective cognitive complaints to dementia: who is at risk?: a systematic review. Am J Alzheimers Dis Other Dement.

[50] Roehr S, Luck T, Pabst A (2017). Subjective cognitive decline is longitudinally associated with lower health-related quality of life. Int Psychogeriatr.

[51] Waldorff FB, Siersma V, Waldemar G (2009). Association between subjective memory complaints and health care utilisation: a three-year follow up. BMC Geriatr.

[52] Bhome R, Berry AJ, Huntley JD, Howard RJ (2018). Interventions for subjective cognitive decline: systematic review and meta-analysis. BMJ Open.

[53] Livingston G, Sommerlad A, Orgeta V (2017). Dementia prevention, intervention, and care. Lancet..

