# Supplementary material for: Estimating prevalence of subjective cognitive decline in and across international cohort studies of aging: a COSMIC study
Source: Alzheimers Res Ther. 2020 Dec 18;12:167. doi: 10.1186/s13195-020-00734-y (PMC7749505; doi:10.1186/s13195-020-00734-y)
Supplement: Supplementary file 1 — Additional file 1. [file 13195_2020_734_MOESM1_ESM.docx]

**Supplement**

**Estimating prevalence of subjective cognitive decline in and across international cohort studies of aging:**

**a COSMIC study**

INDEX

[1. Tables 2](#_Toc11416466)

Table e-1. Key references for contributing studies ……………………………………………………………………………………………………………… 2

[Table e-2. Dementia diagnosis at baseline 3](#_Toc11416467)

[Table e-3. Selected items forming a bridging item to indicate a self-experienced decline in cognitive capacity across studies. 4](#_Toc11416468)

[Table e-4. Availability of additional items for a self-experienced decline in cognitive capacity 5](#_Toc11416469)

[Table e-5. Instruments used for assessing instrumental activities of daily living (IADL) 7](#_Toc11416470)

[Table e-6. Harmonization protocol for major depressive symptomatology 8](#_Toc11416471)

[Table e-7. Harmonization protocol for major anxiety symptomatology 9](#_Toc11416472)

Table e-8. Study-based prevalence estimates for subjective cognitive decline (SCD; right column) and cumulative frequency estimates for the stepwise application of SCD operationalization criteria according to quantitative harmonization based on studies with multiple items……………………………….............................................................. 10

Table e-9. Screening test items and neuropsychological tests used to represent cognitive domains to establish cognitive functioning for sensitivty analysis in 10 studies with sufficient data availability…………………………………………………………………………………………………………………………………………………………...11

Table e-10. Sub-group specific prevalence estimates for subjective cognitive decline (SCD), based on MMSE-derived cognitive functioning and all four operationalization criteria, using the total sample of all 16 studies…………………………………………………………………………………………………………………………………......12

Table e-11.Study-based prevalence estimates for subjective cognitive decline (SCD; right column) and cumulative frequency estimates for the stepwise application of SCD operationalization criteria according to qualitative harmonization based on studies with at least three out of four cognitive domain scores covering memory, language,

processing speed and executive function…………………………………………………………………………………………………………………………………………....13

Table e-12. Study-based prevalence estimates for subjective cognitive decline (SCD; right column) and cumulative frequency estimates for the stepwise application of SCD operationalization criteria according to quantitative harmonization/IRT based on studies with at least three out of four cognitive domain scores covering memory, language, processing speed and executive function…………………………………………………………………………………………………………………………………………....14

Table e-13. Cross-study total prevalence estimates for subjective cognitive decline (SCD) and cumulative frequency estimates for the stepwise application of SCD operationalization criteria according to different methodological approaches for studies (n = 10) with MMSE scores and cognitive domain scores………………………..…...15

[2. References](#_Toc11416477) ….………….................................... ....…… ……. 16

1. Tables

| **Table e-1. Key references for contributing studies.** | |
| --- | --- |
| **Study** | **Key reference** |
| Atma Jaya Cognitive & Aging Research (ActiveAging) | Turana et al. 2014^1^ |
| Cognitive Function & Ageing Studies (CFAS) | Brayne, McCracken & Matthews 2006^2^ |
| Einstein Aging Study USA (EAS) | Katz et al. 2012^3^ |
| Epidemiology of dementia in Central Africa (EPIDEMCA) | Guerchet et al. 2014^4^ |
| Etude Santé Psychologique et Traitement (ESPRIT) | Ritchie, Carrière, Ritchie, Berr, Artero & Ancelin 2010^5^ |
| Invecchiamento Cerebrale in Abbiategrasso (Invece.Ab) | Guaita et al. 2013^6^ |
| Korean Longitudinal Study on Cognitive Aging and Dementia (KLOSCAD) | Han et al. 2018^7^ |
| Leipzig Longitudinal Study of the Aged (LEILA75+) | Riedel-Heller, Schork, Matschinger & Angermeyer 2000^8^ |
| Long-term Research Grant Scheme - Towards Useful Aging (LRGS-TUA) | Shahar et al. 2015^9^ |
| Maastricht Aging Study (MAAS) | Jolles, van Boxtel, Ponds, Metsmakers & Houx 1998^10^ |
| Monongahela Valley Independent Elders Survey (MoVIES) | Ganguli, Mendelsohn, Lytle & Dodge 1998^11^ |
| Personality and Total Health Through Life Project (PATH) | Anstey et al. 2012^12^ |
| Sasaguri Genkimon Study (SGS) | Narazaki, Nofuji, Honda, Matsuo, Yonemoto, Kumagai 2013^13^ |
| Singapore Longitudinal Ageing Studies II (SLASII) | Feng et al. 2010^14^ |
| Sydney Memory and Ageing Study (SyndeyMAS) | Sachdev et al. 2010^15^ |
| Zaragoza Dementia Depression Project (ZARADEMP) | Lobo et al. 2005^16^ |
| \| **Table e-2. Dementia diagnosis at baseline.** \| \| \| --- \| --- \| \| **Study** \| **Dementia criteria** \| \| ActiveAging \| DSM-IV^17^ \| \| CFAS \| AGECAT^18^; organicity level of O3 \| \| EAS \| DSM-IV \| \| EPIDEMCA \| DSM-IV \| \| ESPRIT \| Standardized interview by a neurologist incorporating cognitive testing, with diagnoses validated by an independent panel of expert neurologists. \| \| Invece.Ab \| DSM-IV \| \| KLOSCAD \| DSM-IV \| \| LEILA75+ \| DSM-IV \| \| LRGS-TUA \| N/A \| \| MAAS \| N/A \| \| MoVIES \| CDR^19^ ≥ 1 \| \| PATH \| DSM-IV \| \| SGS \| Self-reported medical history \| \| SLASII \| DSM-IV \| \| SydneyMAS \| DSM-IV \| \| ZARADEMP \| DSM-IV \| \| Abbreviations: AGECAT: Automated Geriatric Examination for Computer Assisted Taxonomy), CDR = Clinical Dementia Rating; DSM - Diagnostic and Statistical Manual of Mental Disorders; N/A = Not applicable/dementia excluded per study design \| \| | |
|  | |

| **Table e-3. Selected items forming a bridging item to indicate a self-experienced decline in cognitive capacity across studies.** | | | | |
| --- | --- | --- | --- | --- |
| **Study** | **Selected item*** | **Coding**** | **Instrument** | **Item properties** |
| ActiveAging | Do you feel you have more problems with your memory than most? | 1 = yes, 2 = no | GDS^20^ | Memory; relation to others |
| CFAS | Have you ever had any difficulty with your memory? If yes, is that a problem for you? | 0 = no, 1 = yes, moderate, 2 = yes, severe | NSQ | Memory; relation to time: ever |
| EAS | Compared with one year ago, do you have trouble remembering things more often, less often or about the same? | 1 = more often, 2 = less often, 3 = about the same | NSQ | Memory; relation to time: 1 year |
| EPIDEMCA | GMS - Subjective difficulty, i.e., implies memory impairment | 0 = no, 1 = yes | NSQ | Memory |
| ESPRIT | Are you usually experiencing any of the following symptoms: Forgetful in everyday activities? | 0 = no, 1 = yes | NSQ | Memory |
| Invece.Ab | Do you think to have memory problems? | 0 = no, 1 = yes | NSQ | Memory |
| KLOSCAD | Do you think that you have a memory problem? | 0 = no, 1 = yes | SMCQ^21,22^ | Memory |
| LEILA75+ | Do have problems with your memory? | 0 = no, 1 = yes | NSQ | Memory |
| LRGS-TUA | Are you having problems with your memory currently? | 0 = no, 1 = yes | GDS | Memory |
| MAAS | Have cognitive failures increased during past 5 years? | 0 = not at all, 1 = very little, 2 = somewhat, 3 = much, 4 = very much | NSQ | Cognition, relation to time: 5 years |
| MoVIES | In general, how good do you feel your memory is for a person your age? | 1 = excellent, 2 = good, 3 = fair, 4 = poor | NSQ | Memory, relation to age |
| PATH | Remembering things that have happened recently | 1 = much improved, 2 = a bit improved, 3 = not much change, 4 = a bit worse, 5 = much worse | IQCODE^22^ | Memory: relation to time: recent; self-administered |
| SGS | Are you sometimes unable to recall date of today/a day? | 1 = yes, 2 = no | NSQ | Memory |
| SLASII | Overall, how would you rate your memory or other mental abilities as compared to earlier period of your life (more than one year ago)? | 1 = much better, 2 = a bit better, 3 = a bit worse, 5 = much worse | NSQ | Memory, cognition: relation to time: >1 year |
| SydneyMAS | Have you noticed difficulties with your memory? | 1 = no, 2 = yes | NSQ | Memory |
| ZARADEMP | Have you had any difficulty with your memory? | 0 = no, 1 = yes | NSQ | Memory |
| Abbreviations: GDS = Geriatric Depression Scale; NSQ = No specified instrument/self-formulated questions; IQCODE = Informant Questionnaire for Cognitive Decline in the Elderly; SMCQ = Subjective Memory Complaints Questionnaire  *measurement approach: population-based; respondent category: self-report; administration mode: interview; **for harmonization, categories were collapsed/dichotomized and/or recoded, if necessary, into 0 = no (absence of a self-experienced decline in cognitive capacity) and 1 = yes (presence of a self-experienced decline in cognitive capacity). Any indication of a self-experienced decline in cognitive capacity was categorized as “1”. | | | | |

| **Table e-4. Availability of additional items for a self-experienced decline in cognitive capacity.** | | | | | | | | | | | | | | | | | |
| --- | --- | --- | --- | --- | --- | --- | --- | --- | --- | --- | --- | --- | --- | --- | --- | --- | --- |
| **Var#** | **Items** | **ActiveAging** | **CFAS** | **EAS** | **EPIDEMCA** | **ESPRIT** | **Invece.Ab** | **KLOSCAD** | **LEILA** | **LRGS-TUA** | **MAAS** | **MoVIES** | **PATH** | **SGS** | **SLASII** | **SydMAS** | **ZARADEMP** |
| 1* | *Bridging item/see Table S2* |  |  |  |  |  |  |  |  |  |  |  |  |  |  |  |  |
| 2 | Have you tended to forget things recently? |  |  |  |  |  |  |  |  |  |  |  |  |  |  |  |  |
| 3 | Difficulty remembering names/things of close people |  |  |  |  |  |  |  |  |  |  |  |  |  |  |  |  |
| 4 | Difficulty remembering where you kept/put things |  |  |  |  |  |  |  |  |  |  |  |  |  |  |  |  |
| 5 | More effort to remember things than used to? |  |  |  |  |  |  |  |  |  |  |  |  |  |  |  |  |
| 6 | In the past year, how often did you have trouble remembering things? |  |  |  |  |  |  |  |  |  |  |  |  |  |  |  |  |
| 7 | Memory worse than 10 years ago |  |  |  |  |  |  |  |  |  |  |  |  |  |  |  |  |
| 8 | Difficulty in remembering new, simple information/5 minutes ago |  |  |  |  |  |  |  |  |  |  |  |  |  |  |  |  |
| 9 | Difficulty in remembering memories/things from a long time ago |  |  |  |  |  |  |  |  |  |  |  |  |  |  |  |  |
| 10 | Difficulty with sums in comparison with past |  |  |  |  |  |  |  |  |  |  |  |  |  |  |  |  |
| 11 | Language difficulties/finding the right word |  |  |  |  |  |  |  |  |  |  |  |  |  |  |  |  |
| 12 | Difficulty with direction/finding way in familiar places |  |  |  |  |  |  |  |  |  |  |  |  |  |  |  |  |
| 13 | Memory is poorer than that of people similar of age |  |  |  |  |  |  |  |  |  |  |  |  |  |  |  |  |
| 14 | Difficulty in remembering a recent event/what happened recently |  |  |  |  |  |  |  |  |  |  |  |  |  |  |  |  |
| 15 | Difficulty remembering a conversation a few days ago |  |  |  |  |  |  |  |  |  |  |  |  |  |  |  |  |
| 16 | Difficulty remembering an appointment made a few days ago |  |  |  |  |  |  |  |  |  |  |  |  |  |  |  |  |
| 17 | Difficulty recognizing familiar people |  |  |  |  |  |  |  |  |  |  |  |  |  |  |  |  |
| 18 | Do you lose objects more often than you did previously |  |  |  |  |  |  |  |  |  |  |  |  |  |  |  |  |
| 19 | Difficulty remembering what to buy when shopping |  |  |  |  |  |  |  |  |  |  |  |  |  |  |  |  |
| 20 | Forget to turn off gas, light, stove, lock the door |  |  |  |  |  |  |  |  |  |  |  |  |  |  |  |  |
| 21 | Difficulty remembering phone numbers of own children/people you call often |  |  |  |  |  |  |  |  |  |  |  |  |  |  |  |  |
| 22 | Difficulty remembering how to use appliances, tools, gadgets |  |  |  |  |  |  |  |  |  |  |  |  |  |  |  |  |
| 23 | Difficulty remembering recipes without looking it up |  |  |  |  |  |  |  |  |  |  |  |  |  |  |  |  |
| 24 | Difficulty remembering your address and telephone number |  |  |  |  |  |  |  |  |  |  |  |  |  |  |  |  |
| 25 | Difficulty remembering what day and month it is |  |  |  |  |  |  |  |  |  |  |  |  |  |  |  |  |
| 26 | Difficulty remembering where things are when put in a place other than usual |  |  |  |  |  |  |  |  |  |  |  |  |  |  |  |  |
| 27 | Difficulty following a conversation |  |  |  |  |  |  |  |  |  |  |  |  |  |  |  |  |
| 28 | Difficulty explaining things |  |  |  |  |  |  |  |  |  |  |  |  |  |  |  |  |
| 29 | Difficulty organizing or planning things |  |  |  |  |  |  |  |  |  |  |  |  |  |  |  |  |
| 30 | Difficulty solving problems |  |  |  |  |  |  |  |  |  |  |  |  |  |  |  |  |
| 31 | Difficulty finding way around unfamiliar places |  |  |  |  |  |  |  |  |  |  |  |  |  |  |  |  |
| 32 | Lose track of what you are doing |  |  |  |  |  |  |  |  |  |  |  |  |  |  |  |  |
| *marked items formed the commensurate measure selected by item response theory modelling | | | | | | | | | | | | | | | | | |

| **Table e-5. Instruments used for assessing instrumental activities of daily living (IADL)** | |
| --- | --- |
| **Study** | **IADL Instruments** |
| ActiveAging | Lawton & Brody IADL Scale^23^ |
| CFAS | Modified Townshend Disability Scale^24^ |
| EAS | Lawton & Brody IADL Scale |
| EPIDEMCA | Adapted Lawton & Brody IADL Scale |
| ESPRIT | Lawton & Brody IADL Scale |
| Invece.Ab | Lawton & Brody IADL Scale |
| KLOSCAD | Disability Assessment of Dementia (DAD)^25^ |
| LEILA75+ | SIDAM-A structured interview for the diagnosis of dementia of the Alzheimer type, multi-infarct dementia and dementias of other aetiology according to ICD-10, DSM-III-R and DSM-IV (SIDAM-B)^26^ |
| LRGS-TUA | Lawton & Brody IADL Scale |
| MAAS | Lawton & Brody IADL Scale |
| MoVIES | Older Americans’ Resources and Services Multidimensional Functional Assessment Questionnaire Center Instrument (OMFAQ)^27^ |
| PATH | Lawton & Brody IADL Scale |
| SGS | Five-item scale for instrumental self-maintenance of the Tokyo Metropolitan Institute of Gerontology Index of Competence (TMIG)^28^ |
| SLASII | Lawton & Brody IADL Scale |
| SydneyMAS | Bayer ADL Scale^29^ |
| ZARADEMP | Lawton & Brody IADL Scale |

| **Table e-6. Harmonization protocol for major depressive symptomatology.** | | |
| --- | --- | --- |
| **Study** | **Current*** | **History*** |
| ActiveAging | GDS-15 score ≥ 11** | N/A |
| CFAS | GMS-AGECAT rating of subcase or clinical case | Diagnosis of depression or manic depression |
| EAS | GDS-15 score ≥ 11 | N/A |
| EPIDEMCA | GMS-AGECAT rating of subcase or clinical case | N/A |
| ESPRIT | 1. Current major depressive episode (MINI neuropsychiatric exam^30^)  2. CES-D^31^ score ≥ 24 | History of major depressive episode (MINI neuropsychiatric exam) |
| Invece.Ab | 1. Use of anti-depressants  2. GDS-15 score ≥ 11  3. Criteria-based diagnosis by physician/ psychologist (including medication, GDS score and CES-D items) | N/A |
| KLOSCAD | 1. Diagnosis with status as “following-up” or “under treatment”  2. NPI^32^ part D  3. Clinical diagnosis of major depressive disorder  4. GDS-K score ≥ 16 (range: 0-30)^33^ | 1. History of depression |
| LEILA75+ | 1. DSM-IV criteria based on structured clinical interview  2. CES-D score ≥ 24 | Self-reported history |
| LRGS-TUA | GDS-15 score ≥ 11 | N/A |
| MAAS | SCL-90^34^ Depression subscale | N/A |
| MoVIES | mCES-D ≥ 5 | N/A |
| PATH | 1. GADS^35^ ≥ 6  2. Use of anti-depressants | Self-reported history and doctor seen |
| SGS | K6^36^ ≥ 5 (combined with anxiety) | N/A |
| SLASII | 1. GDS-15 score ≥ 11  2. Major depressive disorder by SCID | History of depression by antidepressant and report |
| SydneyMAS | 1. GDS-15 score ≥ 11  2. Use of medication/treatment with GP/psychiatrist/psychologist/in hospital | Depression ever diagnosed |
| ZARADEMP | GMS-AGECAT rating of subcase or clinical case | N/A |
| Abbreviations: CES-D = Centre for Epidemiological Studies - Depression Scale; CIRS = Cumulative Illness Rating Scale; DSM-IV = Diagnostic and Statistical Manual of Mental Disorders (4th edition); GADS = Goldberg Anxiety and Depression Scale depression score; GDS-15 = 15 item Geriatric Depression Scale; GDS-K = Korean Geriatric Depression Scale; GMS-AGECAT = Geriatric Mental State - Automated Geriatric Examination for Computer Assisted Taxonomy; GP = general practitioner; ICD-10 = International Classification of Diseases (10th revision); mCES-D = modified Centre for Epidemiological Studies - Depression Scale; K6 =  Kessler Psychological Distress Scale; MINI = Mini International Neuropsychiatric Interview; N/A = not available; NPI = Neuropsychiatric Inventory; SCID = Structured Clinical Interview for DSM-IV; SCL-90 = Symptom Checklist  *For studies with more than one measure, meeting criteria for either was sufficient.  **Items related to memory problems were disregarded if endorsed for all GDS versions across studies. | | |

| **Table e-7. Harmonization protocol for major anxiety symptomatology** | |
| --- | --- |
| **Study** | **Criteria** |
| ActiveAging | N/A |
| CFAS | GMS-AGECAT confidence/severity rating ≥ 3 |
| EAS | BAI^37^ ≥ 19 |
| EPIDEMCA | GMS-AGECAT probable Anxiety |
| ESPRIT | MINI neuropsychiatric exam diagnosis of current anxiety or generalized anxiety disorder |
| Invece.Ab | Use of anxiolytics |
| KLOSCAD | 1. Diagnosis with status “following-up” or “under treatment”  2. NPI part E [scale score=anxiety x severity] |
| LEILA75+ | N/A |
| LRGS-TUA | N/A |
| MAAS | SCL-90 Anxiety Subscale |
| MoVIES | N/A |
| PATH | 1. GADS, anxiety score ≥ 5  2. Anxiety medications taken |
| SGS | K6, anxiety score ≥ 5 |
| SLASII | self-reported history of anxiety, or presence of anxiety symptoms based on NPI |
| SydneyMAS | GADS, anxiety score ≥ 5 |
| ZARADEMP | GMS-AGECAT diagnosis |
| Abbreviation: BAI = Beck Anxiety Scale; GADS = Goldberg Anxiety and Depression Scale depression score; GMS-AGECAT = Geriatric Mental State-Automated Geriatric Examination for Computer Assisted Taxonomy; K6 =  Kessler Psychological Distress Scale; MINI = Mini International Neuropsychiatric Interview; N/A = not available; NPI = Neuropsychiatric Inventory; SCL-90 = Symptom Checklist | |

| **Table e-8. Study-based age- and gender-standardized prevalence estimates for subjective cognitive decline (SCD; right column) and cumulative frequency estimates for the stepwise application of SCD operationalization criteria according to quantitative harmonization/IRT based on studies with additional items for a self-experienced decline in cognitive capacity.** | | | | |
| --- | --- | --- | --- | --- |
| **Study** | **SCD operationalization criteria** | | | |
|  | **Criterion 1**  **Endorsement of a self-experienced decline in cognitive capacity without objective cognitive impairment** | **+ Criterion 2**  **Intact functional ability** | **+ Criterion 3**  **No major depression** | **+ Criterion 4**  **No anxiety disorder** |
|  | % (95%CI) | | | |
| CFAS | 23.53 (22.13-24.93) | 21.62 (20.22-23.01) | 17.83 (16.64-19.02) | 9.40 (8.44-10.36) |
| EAS | 50.33 (44.88-55.78) | 45.84 (40.61-51.06) | 39.05 (34.67-44.33) | 25.64 (18.76-32.52) |
| EPIDEMCA | 57.71 (55.58-59.85) | 54.35 (52.13-56.58) | 31.38 (29.21-33.55) | 28.43 (26.42-30.44) |
| ESPRIT | 31.97 (30.01-33.93) | 29.49 (27.58-31.41) | 24.55 (22.74-26.36) | 18.90 (17.34-20.47) |
| KLOSCAD | 55.59 (54.16-57.02) | 50.61 (49.15-52.08) | 36.00 (34.60-37.41) | 35.84 (34.52-37.16) |
| MoVIES | 30.01 (27.61-32.54) | 27.83 (25.41-30.24) | 24.39 (22.06-26.71) | N/A |
| PATH | 11.93 (10.71-13.14) | 10.98 (9.80-12.16) | 9.82 (8.69-10.95) | 7.84 (6.78-8.90) |
| SLASII | 25.61 (23.48-27.74) | 23.00 (20.94-25.06) | 22.31 (20.28-24.35) | 21.16 (19.32-23.01) |
| SydneyMAS | 46.48 (44.36-48.60) | 43.35 (41.09-45.61) | 36.42 (34.08-38.77) | 29.20 (26.94-31.47) |
| **Total** | **36.52 (35.97-37.08)** | **33.73 (33.18-34.28)** | **27.02 (26.50-27.54)** | **26.07 (25.43-26.72)** |
| Abbreviations: 95% CI = 95% confidence interval; IRT = Item Response Theory; N/A = not available | | | | |

| **Table e-9. Screening test items and neuropsychological tests used to represent cognitive domains to establish cognitive functioning for sensitivity analysis across 10 studies with sufficient data availability.** | | | | |
| --- | --- | --- | --- | --- |
| **Study** | **Memory** | **Language** ^a^ | **Processing Speed** | **Executive Function** |
| ActiveAging | MMSE 3-word list recall | Verbal Fluency Test (Animals) | DigitSpan (Forward) | N/A |
| EAS | Free and Cued Selective Reminding Test | Verbal Fluency Test (Animals) | Trail Making Test A ^b^ | Trail Making Test B (=300s if TMTA≥0 and TMTB missing or >300) |
| ESPRIT | MMSE 3-word list recall | Verbal Fluency Test (Animals; 30 seconds) | Trail Making Test A | Trail Making Test B ^b^ |
| Invece.Ab | RAVLT trial 7 (15 min delay) | Verbal Fluency Test (Mean of Colours, Animals, Fruits, Cities; each 120s) | Trail Making Test A | Trail Making Test B |
| KLOSCAD | CERAD 10-word list recall test | Verbal Fluency Test (Animals) | Trail Making Test A (360 seconds) | Trail Making Test B (360 seconds) |
| LRGS-TUA | RAVLT trial 7 (15 min delay) | MoCA 3 language items | DigitSpan (Forward) | DigitSymb |
| MoVIES | CERAD 10-word list recall test | Verbal Fluency Test (Animals) | Trail Making Test A | Trail Making Test B |
| PATH | California Verbal Learning Test (recall of first list) | N/A | Trail Making Test A | Trail Making Test B |
| SLASII | RAVLT trial 7 | N/A | Color Trails Test 1 | Color Trails Test 2 |
| SyndeyMAS | RAVLT trial 7 | Verbal Fluency Test (Animals) | Trail Making Test A | Trail Making Test B |

Abbreviations (tests): CERAD, Consortium to Establish a Registry for Alzheimer’s Disease neuropsychological assessment battery; MMSE, Mini-Mental State Examination; MoCA, Montreal Cognitive Assessment; RAVLT, Rey Auditory Verbal Learning Test;

^a^ Semantic fluency test: category words generated in 60s unless otherwise indicated.

^b^ 300 seconds unless otherwise indicated.

| **Table e-10. Sub-group specific prevalence estimates for subjective cognitive decline (SCD), based on MMSE-derived cognitive functioning and all four operationalization criteria, using the total sample of all 16 studies.** | | | | |
| --- | --- | --- | --- | --- |
|  | **Qualitative approach** | | **Quantitative approach/IRT** | |
|  | **% (95%CI)** | ***Χ*²(df), *p*-Value** | **% (95%CI)** | **p-Value** |
| **Age** |  |  |  |  |
| 60 - <65 | 29.87 (28.25-31.48) | 83.76 (5), p <.001 | 27.37 (25.79-28.94) |  |
| 65 - <70 | 22.70 (21.66-23.74) |  | 23.18 (22.14-24.23) |  |
| 70 - <75 | 21.93 (21.01-22.86) |  | 24.52 (23-56-25.48) |  |
| 75 - <80 | 24.62 (23.28-25.96) |  | 27.90 (26.51-29.29) |  |
| 80 - <85 | 24.30 (22.51-26.09) |  | 28.05 (26.18-29.92) |  |
| 85+ | 22.96 (20.73-25.19) |  | 28.05 (25.68-30.43) | 51.31 (5), p .<001 |
| **Sex** |  |  |  |  |
| Male | 25.37 (24.53-26.21) |  | 26.56 (25.71-27.42) |  |
| Female | 22.72 (22.03-23.41) | 23.27 (1), p <.001 | 24.92 (24.21-25.63) | 8.54 (1), p = .003 |
| **Education** |  |  |  |  |
| Pre- / primary | 26.40 (25.25-27.54) |  | 29.04 (27.85-30.22) |  |
| Secondary / middle | 24.19 (23.28-25.09) |  | 24.97 (24.05-26.88) |  |
| Post- / secondary upper | 20.54 (19.55-21.53) |  | 22.74 (21.71-23.76) |  |
| Tertiary | 23.02 (21.50-24.55) | 62.05 (3), p <.001 | 26.35 (24.73-27.97) | 68.37 (3), p <.001 |
| **Ethnicity** |  |  |  |  |
| Asian people | 28.38 (27.46-29.30) |  | 27.16 (26.24-28.07) |  |
| Black African people | 23.74 (21.81-25.66) |  | 28.27 (26.21-30.26) |  |
| White people | 20.91 (20.15-21.66) | 165.40 (2), p < .001 | 24.27 (23.48-25.06) | 29.39 (2), p < .001 |
| **Country income** |  |  |  |  |
| LIC | 24.85 (22.11-27.85) |  | 29.34 (26.43-32.25) |  |
| LMIC | 23.50 (22.95-24.05) |  | 27.45 (24.63-30.26) |  |
| HIC | 22.84 (20.11-25.56) | 0.99 (2), p = .611 | 25.05 (24.49-25.61) | 9.56 (2), p = .008 |
| **Decade** |  |  |  |  |
| < 1999 | 19.70 (18.78-20.62) |  | 22.37 (21.40-23.33) |  |
| 2000-2009 | 21.61 (15.69-27.34) |  | 24.50 (23.08-25.92) |  |
| > 2009 | 26.59 (25.84-27.35) | 203.93 (2), p < .001 | 26.61 (25.85-27.36) | 47.77 (2), p <.001 |
| Abbreviations: 95% CI = 95% confidence interval; IRT = Item Response Theory; HIC = high-income country; LIC = low-income country; LMIC = low-middle-income country; MMSE = Mini-Mental Status Examination | | | | |

| **Table e-11. Study-based prevalence estimates for subjective cognitive decline (SCD; right column) and cumulative frequency estimates for the stepwise application of SCD operationalization criteria according to qualitative harmonization based on studies with at least three out of four cognitive domain scores covering memory, language, processing speed and executive function.** | | | | |
| --- | --- | --- | --- | --- |
| **Study** | **SCD operationalization criteria** | | | |
|  | **Criterion 1**  **Endorsement of a self-experienced decline in cognitive capacity without objective cognitive impairment** | **+ Criterion 2**  **Intact functional ability** | **+ Criterion 3**  **No major depression** | **+ Criterion 4**  **No anxiety disorder** |
|  | % (95%CI) | | | |
| ActiveAging | 29.39 (22.29-36.48) | 26.71 (19.80-33.62) | 26.68 (19.77-33.60) | N/A |
| EAS | 14.70 (11.63-17.77) | 12.80 (10.04-15.56) | 14.41 (10.43-18.39) | 12.00 (5.96-18.05) |
| ESPRIT | 16.38 (14.74-18.01) | 15.40 (13.81-17.00) | 12.35 (10.90-13.81) | 10.08 (8.74-11.42) |
| Invece.Ab | 18.94 (13.95-23.93) | 18.06 (13.07-23.04) | 16.01 (11.32-20.69) | 11.94 (9.47-14.41) |
| KLOSCAD | 62.17 (60.71-63.63) | 57.74 (56.24-59.24) | 42.89 (41.38-44.39) | 42.83 (41.32-44.35) |
| LRGS-TUA | 51.06 (48.23-53.88) | 47.58 (44.75-50.42) | 47.11 (44.31-49.92) | N/A |
| MoVIES | 31.13 (28.60-33.66) | 29.46 (26.97-31.95) | 26.35 (23.91-28.78) | N/A |
| PATH | 9.71 (8.57-10.84) | 8.88 (7.79-9.98) | 7.95 (6.89-9.01) | 6.71 (5.71-7.72) |
| SLASII | 12.07 (9.88-14.27) | 11.17 (9.08-13.27) | 10.96 (8.89-13.02) | 10.71 (8.68-12.73) |
| SyndeyMAS | 41.78 (39.10-44.46) | 38.40 (35.57-41.23) | 32.59 (29.72-35.46) | 29.35 (26.35-32.34) |
| **Total** | **39.48 (38.71-40.25)** | **36.98 (36.22-37.51)** | **31.27 (30.52-32.01)** | **28.58 (27.74-29.43)** |
| Abbreviations: 95% CI = 95% confidence interval; N/A = not available | | | | |

| **Table e-12. Study-based prevalence estimates for subjective cognitive decline (SCD; right column) and cumulative frequency estimates for the stepwise application of SCD operationalization criteria according to quantitative harmonization/IRT based on studies with at least three out of four cognitive domain scores covering memory, language, processing speed and executive function.** | | | | |
| --- | --- | --- | --- | --- |
| **Study** | **SCD operationalization criteria** | | | |
|  | **Criterion 1**  **Endorsement of a self-experienced decline in cognitive capacity without objective cognitive impairment** | **+ Criterion 2**  **Intact functional ability** | **+ Criterion 3**  **No major depression** | **+ Criterion 4**  **No anxiety disorder** |
|  | % (95%CI) | | | |
| ActiveAging | 29.38 (22.28-36.48) | 26.71 (19.79-33.62) | 26.68 (19.77-33.60) | N/A |
| EAS | 43.28 (38.15-48.40) | 38.90 (34.10-43.70) | 37.68 (33.33-42.04) | 26.39 (19.89-32.89) |
| ESPRIT | 28.42 (26.48-30.36) | 26.71 (24.80-28.62) | 22.26 (20.46-24.07) | 18.07 (16.58-19.76) |
| Invece.Ab | 18.98 (13.96-23.99) | 18.09 (13.08-23.10) | 16.03 (11.32-20.74) | 11.96 (9.47-14.44) |
| KLOSCAD | 54.02 (52.52-55.52) | 50.25 (48.73-51.76) | 36.68 (35.21-38.15) | 36.66 (35.18-38.14) |
| LRGS-TUA | 51.06 (48.23-53.90) | 47.59 (44.75-50.42) | 47.11 (44.30-49.93) | N/A |
| MoVIES | 27.20 (24.76-29.64) | 25.71 (23.30-28.12) | 22.89 (20.56-25.22) | N/A |
| PATH | 12.56 (11.30-13.82) | 11.49 (10.27-12.71) | 10.39 (9.21-11.57) | 8.67 (7.55-9.79) |
| SLASII | 23.60 (20.78-26.41) | 22.01 (19.26-24.76) | 21.35 (18.65-24.05) | 20.33 (17.72-22.94) |
| SyndeyMAS | 44.73 (42-30-47.16) | 41.66 (39.05-44.26) | 35.05 (32.33-37.77) | 31.82 (28.97-34.67) |
| **Total** | **43.24 (42.47-44.02)** | **40.55 (39.78-41.33)** | **34.65 (33.89-35.41)** | **29.56 (28.71-30.40)** |
| Abbreviations: 95% CI = 95% confidence interval; IRT = Item Response Theory; N/A = not available | | | | |

| **Table e-13. Cross-study total prevalence estimates for subjective cognitive decline (SCD) and cumulative frequency estimates for the stepwise application of SCD operationalization criteria according to different methodological approaches for studies (n = 10) with MMSE scores and cognitive domain scores.** | | | | | |
| --- | --- | --- | --- | --- | --- |
|  | **SCD operationalization criteria** | | | | |
|  | **Criterion 1**  **Endorsement of a self-experienced decline in cognitive capacity without objective cognitive impairment** | | **+ Criterion 2**  **Intact functional ability** | **+ Criterion 3**  **No major depression** | **+ Criterion 4**  **No anxiety disorder** |
|  | % (95%CI) | | | | |
| **Qualitative approach** |  |  |  |  |  |
| MMSE | 38.59 (37.90-39.28) | | 35.77 (35.09-36.46) | 29.92 (29.26-30.58) | 27.11 (26.37-27.84) |
| Cognitive domains | 39.48 (38.71-40.25) | | 36.98 (36.22-37.51) | 31.27 (30.52-32.01) | 28.58 (27.74-29.43) |
| **Quantitative approach/IRT** |  | |  |  |  |
| MMSE | 42.69 (41.97-43.39) | | 39.63 (38.94-40.33) | 33.65 (32.98-34.33) | 28.76 (28.01-29.50) |
| Cognitive domains | 43.24 (42.47-44.02) | | 40.55 (39.78-41.33) | 34.65 (33.89-35.41) | 29.56 (28.71-30.40) |
| Abbreviations: 95% CI = 95% confidence interval; IRT = Item Response Theory; MMSE = Mini-Mental Status Examination, N/A = not available | | | | | |

**2. References**

1. Turana Y, Ranakusuma TAS, Purba JS, Amir N, Ahmad SA, Machfoed MH, et al. Enhancing Diagnostic Accuracy of aMCI in the Elderly: Combination of Olfactory Test, Pupillary Response Test, BDNF Plasma Level, and APOE Genotype. *Int J Alzheimers Dis*. 2014;2014:9.

2. Brayne C, McCracken C, Matthews FE. Cohort profile: the Medical Research Council Cognitive Function and Ageing Study (CFAS). *Int J Epidemiol*. 2006;35(5):1140–1145.

3. Katz MJ, Lipton RB, Hall CB, Zimmerman ME, Sanders AE, Verghese J, et al. Age-specific and sex-specific prevalence and incidence of mild cognitive impairment, dementia, and Alzheimer dementia in blacks and whites: a report from the Einstein Aging Study. *Alzheimer Dis Assoc Disord*. 2012;26(4):335–343.

4. Guerchet M, Mbelesso P, Ndamba-Bandzouzi B, Pilleron S, Desormais I, Lacroix P, et al. Epidemiology of dementia in Central Africa (EPIDEMCA): protocol for a multicentre population-based study in rural and urban areas of the Central African Republic and the Republic of Congo. *Springerplus*. 2014;3(1):338.

5. Ritchie K, Carriere I, Ritchie CW, Berr C, Artero S, Ancelin M-L. Designing prevention programmes to reduce incidence of dementia: prospective cohort study of modifiable risk factors. *BMJ*. 2010;341:c3885.

6. Guaita A, Colombo M, Vaccaro R, Fossi S, Vitali SF, Forloni G, et al. Brain aging and dementia during the transition from late adulthood to old age: design and methodology of the “Invece.Ab” population-based study. *BMC Geriatr*. 2013;13:98.

7. Han JW, Kim TH, Kwak KP, Kim K, Kim BJ, Kim SG, et al. Overview of the Korean Longitudinal Study on Cognitive Aging and Dementia. *Psychiatry Investig*. 2018;15(8):767–774.

8. Riedel-Heller SG, Schork A, Matschinger H, Angermeyer MC. Recruitment Procedures and Their Impact on the Prevalence of Dementia. *Neuroepidemiology*. 2000;19(3):130–140.

9. Shahar S, Omar A, Vanoh D, Hamid TA, Mukari SZ, Din NC, et al. Approaches in methodology for population-based longitudinal study on neuroprotective model for healthy longevity (TUA) among Malaysian Older Adults. *Aging Clin Exp Res*. 2016;28(6):1089–1104.

10. Jolles J, van Boxtel MP, Ponds RW, Metsemakers JF, Houx PJ. The Maastricht aging study (MAAS). The longitudinal perspective of cognitive aging. *Tijdschr Gerontol Geriatr*. 1998;29(3):120–129.

11. Ganguli M, Mendelsohn A, Lytle M, Dodge H. A follow-up comparison of study participants and refusers within a rural elderly population. *J Gerontol A Biol Sci Med Sci*. 1998;53(6):M465–70.

12. Anstey KJ, Christensen H, Butterworth P, Easteal S, Mackinnon A, Jacomb T, et al. Cohort profile: the PATH through life project. *Int J Epidemiol*. 2012;41(4):951–960.

13. Narazaki K, Nofuji Y, Honda T, Matsuo E, Yonemoto K, Kumagai S. Normative data for the montreal cognitive assessment in a Japanese community-dwelling older population. *Neuroepidemiology*. 2013;40(1):23–29.

14. Feng L, Chong MS, Lim WS, Lee TS, Collinson SL, Yap P, Ng TP. Metabolic syndrome and amnestic mild cognitive impairment: Singapore Longitudinal Ageing Study-2 findings. *J Alzheimers Dis*. 2013;34(3):649–657.

15. Sachdev PS, Brodaty H, Reppermund S, Kochan NA, Trollor JN, Draper B, et al. The Sydney Memory and Ageing Study (MAS): methodology and baseline medical and neuropsychiatric characteristics of an elderly epidemiological non-demented cohort of Australians aged 70-90 years. *Int Psychogeriatr*. 2010;22(8):1248–1264.

16. Lobo A, Saz P, Marcos G, Día JL, De-la-Cámara C, Ventura T, et al. The ZARADEMP Project on the incidence, prevalence and risk factors of dementia (and depression) in the elderly community: II. Methods and first results. *Eur J Psychiatry*. 2005;19:40–54.

17. *Diagnostic and statistical manual of mental disorders: DSM-IV:* Fourth edition. Washington, DC: American Psychiatric Association; 1994.

18. Dewey ME, Copeland JRM. Computerized psychiatric diagnosis in the elderly: AGECAT. *J Microcomput Appl*. 1986;9(2):135–140.

19. Morris JC. Clinical dementia rating: a reliable and valid diagnostic and staging measure for dementia of the Alzheimer type. *Int Psychogeriatr*. 1997;9 Suppl 1:173–176; discussion 177–178.

20. Sheikh JI, Yesavage JA. Geriatric Depression Scale (GDS): Recent evidence and development of a shorter version. *Clin Gerontol.* 1986;5(1-2):165–173.

21. Youn JC, Kim KW, Lee DY, Jhoo JH, Lee SB, Park JH, et al. Development of the Subjective Memory Complaints Questionnaire. *Dement Geriatr Cogn Disord*. 2009;27(4):310–317.

22. Jorm AF, Jacomb PA. The Informant Questionnaire on Cognitive Decline in the Elderly (IQCODE): socio-demographic correlates, reliability, validity and some norms. *Psychol Med*. 1989;19(4):1015-1022.

23. Lawton MP, Brody EM. Assessment of Older People: Self-Maintaining and Instrumental Activities of Daily Living1. *Gerontologist*. 1969;9(3_Part_1):179–186.

24. Townsend P. *Poverty in the United Kingdom: A survey of household resources and standards of living.* Repr. Harmondsworth: Penguin; 1983.

25. Gelinas I, Gauthier L, McIntyre M, Gauthier S. Development of a functional measure for persons with Alzheimer’s disease: the disability assessment for dementia. *Am J Occup Ther*. 1999;53(5):471–481.

26. Zaudig M, Mittelhammer J, Hiller W, et al. SIDAM—A structured interview for the diagnosis of dementia of the Alzheimer type, multi-infarct dementia and dementias of other aetiology according to ICD-10 and DSM-III-R. *Psychol Med*. 1991;21(1):225–236.

27. Fillenbaum GG, Smyer MA. The development, validity, and reliability of the OARS multidimensional functional assessment questionnaire. *J Gerontol*. 1981;36(4):428–434.

28. Koyano W, Shibata H, Nakazato K, Haga H, Suyama Y. Measurement of competence: reliability and validity of the TMIG Index of Competence. *Arch Gerontol Geriatr*. 1991;13(2):103–116.

29. Hindmarch I, Lehfeld H, de Jongh P, Erzigkeit H. The Bayer activities of daily living scale (B-ADL). *Dement Geriatr Cogn Disord*. 1998; *9*(Suppl. 2):20–26.

30. Sheehan DV, Lecrubier Y, Sheehan KH, Amorim P, Janavs J, Weiller E, et al. The Mini-International Neuropsychiatric Interview (M.I.N.I.): the development and validation of a structured diagnostic psychiatric interview for DSM-IV and ICD-10. *J Clin Psychiatry*. 1998;59 Suppl 20:22–33;quiz 34–57.

31. Radloff LS. The CES-D scale: A self-report depression scale for research in the general population. *Appl Psychol Meas*. 1977;1(3):385–401.

32. Cummings JL, Mega M, Gray K, Rosenberg-Thompson S, Carusi DA, Gornbein J. The Neuropsychiatric Inventory: comprehensive assessment of psychopathology in dementia. *Neurology*. 1994;44(12):2308–2314.

33. Choe JY, Youn JC, Park JH, Park IS, Jeong JW, Lee WH, et al. The Severe Cognitive Impairment Rating Scale--an instrument for the assessment of cognition in moderate to severe dementia patients. *Dement Geriatr Cogn Disord* 2008;25:321–328.

34. Derogatis LR. *SCL 90 R Administration, Scoring and Procedures Manual II for the Revised Version and Other Instruments of the Psychopathology Rating Scale Series:* Clinical Psychometric Research; 1986.

35. Goldberg DP, Bridges K, Duncan-Jones P, Grayson D. Dimensions of neuroses seen in primary-care settings. *Psychol Med*. 1987;17(2):461–470.

36. Kessler RC, Andrews G, Colpe LJ, Hirpi E, Mroczek DK, Normand SL, et al. Short screening scales to monitor population prevalences and trends in non-specific psychological distress. *Psychol Med*. 2002;32(6):959–976.

37. Beck AT, Epstein N, Brown G, Steer RA. An inventory for measuring clinical anxiety: psychometric properties. *JCCP*. 1988;56(6):893.
